# Complete genome analysis of *Bacillus velezensis* TS5 and its potential as a probiotic strain in mice

**DOI:** 10.3389/fmicb.2023.1322910

**Published:** 2023-12-06

**Authors:** Benhao Chen, Yi Zhou, Lixiao Duan, Xuemei Gong, Xingmei Liu, Kangcheng Pan, Dong Zeng, Xueqin Ni, Yan Zeng

**Affiliations:** ^1^Animal Microecology Institute, College of Veterinary Medicine, Sichuan Agricultural University, Chengdu, China; ^2^Engineering Research Center of Southwest Animal Disease Prevention and Control Technology, Ministry of Education of the People’s Republic of China, Chengdu, China

**Keywords:** probiotic, *Bacillus velezensis* TS5, complete genome, cellulose, digestive enzyme activity

## Abstract

**Introduction:**

In recent years, a large number of studies have shown that *Bacillus velezensis* has the potential as an animal feed additive, and its potential probiotic properties have been gradually explored.

**Methods:**

In this study, Illumina NovaSeq PE150 and Oxford Nanopore ONT sequencing platforms were used to sequence the genome of *Bacillus velezensis* TS5, a fiber-degrading strain isolated from Tibetan sheep. To further investigate the potential of *B. velezensis* TS5 as a probiotic strain, in vivo experiments were conducted using 40 five-week-old male specific pathogen-free C57BL/6J mice. The mice were randomly divided into four groups: high fiber diet control group (H group), high fiber diet probiotics group (HT group), low fiber diet control group (L group), and low fiber diet probiotics group (LT group). The H and HT groups were fed high-fiber diet (30%), while the L and LT groups were fed low-fiber diet (5%). The total bacteria amount in the vegetative forms of *B. velezensis* TS5 per mouse in the HT and LT groups was 1 × 10^9^ CFU per day, mice in the H and L groups were given the same volume of sterile physiological saline daily by gavage, and the experiment period lasted for 8 weeks.

**Results:**

The complete genome sequencing results of *B. velezensis* TS5 showed that it contained 3,929,788 nucleotides with a GC content of 46.50%. The strain encoded 3,873 genes that partially related to stress resistance, adhesion, and antioxidants, as well as the production of secondary metabolites, digestive enzymes, and other beneficial nutrients. The genes of this bacterium were mainly involved in carbohydrate metabolism, amino acid metabolism, vitamin and cofactor metabolism, biological process, and molecular function, as revealed by KEGG and GO databases. The results of mouse tests showed that *B. velezensis* TS5 could improve intestinal digestive enzyme activity, liver antioxidant capacity, small intestine morphology, and cecum microbiota structure in mice.

**Conclusion:**

These findings confirmed the probiotic effects of *B. velezensis* TS5 isolated from Tibetan sheep feces and provided the theoretical basis for the clinical application and development of new feed additives.

## 1 Introduction

The International Scientific Association for Probiotics and Prebiotics (ISAPP) defined probiotics as “live microorganisms that, when administered in adequate amounts, confer a health benefit on the host” (Hill et al., 2014). Probiotics are commonly used to improve host health and maintain the balance of intestinal flora. Commonly used bacterial probiotics are derived mainly from *Lactobacillus*, *Bifidobacterium*, and *Bacillus* (Vera-Santander et al., 2023). Among them, *Bacillus* probiotics have sporulation ability and are more suitable for processing, storage, and survival through the gastrointestinal tract (Lu et al., 2022). Many studies have reported that *Bacillus* can interact with the host at multiple levels, such as secreting antibacterial substances and a variety of digestive enzymes, improving the structure of intestinal flora, regulating immunity, etc., thus exerting its probiotic properties (Du et al., 2018; Hu et al., 2018; Zou et al., 2022; Iqbal et al., 2023). All of these properties and their long shelf life make *Bacillus* direct feeding microbials (DFM) strains and their endospores an ideal feed supplement (Bahaddad et al., 2023). Probiotics strains of *Bacillus* benefit the health of piglets and broilers and help reduce the misuse of direct antibiotics in feed (Luise et al., 2022). In addition, the research results by Bahaddad et al. (2023) also confirmed that *Bacillus* can be used as a direct feed microbial addition for monogastric animals.

*Bacillus velezensis* was first and identified in the river Velez in Málaga, southern Spain (Ruiz-García et al., 2005). This strain is easy to isolated and cultured, and it exists widely in nature, making it a potential probiotic candidate. Due to the absence of toxigenic potential and aminoglycoside production capacity, *Bacillus velezensis* was recommended for Qualified Presumption of Safety (QPS) status in 2020 by European Food Safety Authority (EFSA) (Koutsoumanis et al., 2020). Previous studies have reported the usefulness of *Bacillus velezensis* as a probiotic in the aquaculture (Zhang et al., 2019; Wu et al., 2021; Monzón-Atienza et al., 2022), and it has the potential to promote plant growth and inhibit plant pathogenic fungi (Tzipilevich et al., 2021; Dong et al., 2023; Thanh Tam et al., 2023). Khalid et al. (2021) reviewed the potential properties of *Bacillus velezensis* as a probiotic in the animal feed industry, which is expected to become a candidate probiotic in this industry. Complete genome sequence analysis can effectively identify potential characteristics of probiotics and enhance our understanding of the relationship between genotype and phenotype (Huang et al., 2022). It has been shown that the genomes of *Bacillus velezensis* from different sources, such as spontaneously fermented coconut water (Dhanya Raj et al., 2023), fermented kimchi (Heo et al., 2021), and soil (Pereira et al., 2019), have been well characterized, revealing the reasons for its probiotic properties. In addition, an increasing number of studies suggest that probiotics require animal functional experiments to verify their probiotic properties. Among these experiments, rodents such as mice (Cao et al., 2023), rats (Elkholy et al., 2023), and rabbits (Hou et al., 2023) are the most widely used in clinical practice.

The *Bacillus velezensis* TS5 isolated from Tibetan sheep feces is a potential probiotic strain with *in vitro* probiotic properties. This study aimed to analyze the complete genome sequencing information of this strain and evaluate its digestive promotion and antioxidant effects on mice, including its effects on growth performance, blood routine, small intestine morphology, antioxidant capacity, intestinal digestive enzyme activity, and cecum microbiota.

## 2 Materials and methods

### 2.1 Bacterial strains

The *Bacillus velezensis* TS5 strain was isolated from Tibetan sheep feces and provided by the Animal Microecology Research Center of Sichuan Agricultural University. The bacteria is stored in the Chinese Typical Culture Preservation Center (CCTCC NO: M2023345).

### 2.2 Complete genome sequencing and analysis of bacterial

The genomic DNA of *Bacillus velezensis* TS5 was extracted using the TIANamp Bacteria DNA kit (DP302). The quality and concentration of the extracted DNA were determined using 1% agarose gel electrophoresis and a nucleic acid quantizer. The second and third generations of complete genome sequencing for strain TS5 were conducted using the Illumina NovaSeq PE150 and Oxford Nanopore ONT sequencing platforms, respectively. Libraries were constructed from the samples and sequenced to obtain second-generation data. Quality control of the raw data was performed using FastQC (Patel and Jain, 2012). The third-generation single-molecule sequencing data was assembled using Unicycle and Flye software to obtain contig sequences. To correct the second-generation high-quality data for the third-generation contig results, pilon (Walker et al., 2014) software was used. Finally, the contigs were spliced to obtain a complete sequence.

To construct a phylogenetic tree of multiple site sequence fragments of isolated strains and standard strains, AutoMLST webserver and MEGA X software were used (Alanjary et al., 2019). Additionally, the GGDC (The Genome to Genome Distance Calculator) webserver (Meier-Kolthoff et al., 2022) and the Average Nucleotide Identity (ANI) calculator software (Yoon et al., 2017) on the EzBioCloud platform were utilized to analyze the DNA-DNA Hybridization (DDH) and ANI between strain TS5 and the standard strain. To predict gene islands in the genome of *Bacillus velezensis* TS5, Island Viewer 4 was utilized (Bertelli et al., 2017). Secondary metabolites analysis was performed on genomic data of isolated strains using anti SMASH 6.0 (Blin et al., 2021). Bacterial genomic information was annotated in the Gene Ontology (GO) (Conesa and Götz, 2008), Kyoto Encyclopedia of Genes and Genomes (KEGG) (Moriya et al., 2007), evolutionary genealogy of genes: Non-supervised Orthologous Groups (eggNOG) (Galperin et al., 2015), Swiss-Prot (Pedruzzi et al., 2015), and Carbohydrate-Active Enzymes Database (CAZy) (Lombard et al., 2014) using various tools. Mining candidate genes related to probiotic properties in the genome of *Bacillus velezensis* TS5 based on the above annotation results. The CGView software was then employed to draw a genome circle diagram (Stothard and Wishart, 2005), while the genome sequences were uploaded to the NCBI database with an accession number of CP125654.1.

The BLAST software was used to predict the presence of antibiotic resistance related genes in the genome of *Bacillus velezensis* TS5 in the Comprehensive Antibiotic Resistance Database (CARD) (McArthur et al., 2013). The BLAST alignment parameter *E*-value was set to 1e^–6^, and the consistency of amino acid sequences was above 45%. The ratio of sequence alignment length to sequence length was not less than 70%. The ResFinder 4.1 was used to identify the acquired antibiotic resistance genes (Verschuuren et al., 2022). The BLAST software was used to compare the protein sequences encoded by genes with the amino acid sequences (Set A) in the Virulence Factors of Pathogenic Bacteria (VFDB) database to predict the virulence factor related genes in the genome of *Bacillus velezensis* TS5 (Altschul et al., 1990; Chen et al., 2016). The BLAST alignment parameter *E*-value was set to 1e^–5^, and the consistency of amino acid sequences was over 60%. The ratio of the length of the sequence alignment to the length of the corresponding virulence factor related gene sequence was not less than 70%, and the gap length of the alignment was less than 10% of the sequence alignment length. The Virulence Finder (V2.0.3) was used to predict the presence of virulence genes in the genome of *Bacillus velezensis* TS5 (Joensen et al., 2014).

### 2.3 Animal experimental design

A total of 40 five-week-old male specific pathogen free C57BL/6J mice (weighing 18 ± 1 g) were purchased from Beijing Sibeifu Biotechnology Co., Ltd (China). The mice were randomly divided into four groups: high fiber diet control group (H group), high fiber diet probiotics group (HT group), low fiber diet control group (L group), and low fiber diet probiotics group (LT group), 10 mice per group. Mice in the H and HT groups were fed a high-fiber diet containing 30% cellulose, while those in the L and LT groups were fed a low-fiber diet containing 5% cellulose. Each mouse in the HT and LT groups received 0.2 mL vegetative form of *Bacillus velezensis* TS5 solution at a concentration of 5 × 10^9^ CFU/mL per day, while those in the H and L groups were given 0.2 mL of sterile saline (0.9%). The experimental period was 8 weeks, during which the mental and eating state of the mice were observed every day, and the morbidity, mortality, and feed consumption were recorded. The body weight of each mouse was also recorded regularly every week. The mouse experiments were carried out in the Animal Microecology Research Center of Sichuan Agriculture University, and all experimental procedures adhered to the guidelines for the feeding and use of experimental animals approved by the Committee of Sichuan Agriculture University (SYXKchuan2019-187).

### 2.4 Blood routine testing

At the conclusion of the experimental, blood was collected from the mouse eyeball using tweezers and placed in an anticoagulation tube containing Ethylene Diamine Tetraacetie Acid (EDTA). The content of white blood cells, neutrophils, lymphocytes, monocyte, red blood cells, platelets, and hemoglobin content were immediately measured by a fully automated blood analyzer (BC-5000 Vet, Mindray medical international limited).

### 2.5 HE staining

The jejunum, ileum, liver, kidney and testes tissues of three mice in each group were washed with PBS (pH 7.4) solution and fixed with 4% Paraformaldehyde solution. The tissues were then dehydrated, embedded, sectioned, stained with hematoxylin and eosin, and sealed. The tissues sections were observed under a microscope and photographed. Villus height, crypt depth and villus/crypt (V/C) ratio of the small intestinal tissue were measured and analyzed.

### 2.6 Liver antioxidant testing

Mouse livers were subjected to liquid nitrogen freezing and stored at −80°C for subsequent analysis. The activity of liver catalase (CAT), dismutase (SOD), malondialdehyde (MDA), glutathione peroxidase (GSH Px), and total antioxidant capacity (T-AOC) were determined using ammonium molybdate (A007-1-1), water-soluble tetrazolium salt-1 (WST-1) (A001-3), colorimetry (A003-2), dithiodinitrobenzoic acid (A005-1), and 2,2′-Azino-bis(3-ethylbenzothiazoline-6-sulfonic acid) (ABTS) (A015-2-1) methods, respectively. The test kits were obtained from Nanjing Jiancheng Biotechnology Research Institute and the procedures followed the instructions provided by the kit.

### 2.7 Detection of intestinal digestive enzyme activity

Weighed 0.10 g of intestinal contents and added 0.9 mL of sterile normal saline (0.9%). Homogenized the mixture and centrifuged it at 8000 rpm for 10 min at 4°C. After collecting the supernatant, added 9 mL of sterile normal saline (0.9%) and mixed the resulting solution as crude enzyme solution.

The activities of β-glucosidase, endoglucanase, exoglucanase, filter paper enzyme, and xylanase were determined using 1% salicylic solution, 1% sodium carboxymethyl cellulose solution, 0.05 g absorbent cotton, 0.05 g starch free filter paper, and 1% xylan solution as enzyme reaction substrates [23], respectively. The activities of cellulase and hemicellulase were measured by incubating 0.5 mL of crude enzyme solution and a special substrate in citrate acid buffer (pH 4.8) at 50°C for 30 min (Budihal et al., 2016). The reducing sugar formed after incubation was estimated by the 3, 5-dinitrosalicylic acid method (Miller, 1959). The enzyme activity unit (U) represents the amount of enzyme that produce 1 μg of reducing sugar from a 1.0 g sample hydrolyzing the substrate for 1.0 min. The mass of reducing sugar of the measuring and blank control was recorded as A1 and A0, respectively. The activities of cellulase and hemicellulose were calculated using the following formula:


(1) $$\text{X}=\frac{\left(A\times 1000\times n\right)}{\left(V\times t\right)}$$

In formula (1), X represents the activity of cellulase and hemicellulose, expressed in U/g. A represents the yield of reducing sugar (A1-A0), expressed in mg. 1000 is the conversion factor, 1 mmol = 1000 μmol. “n” represents the dilution ratio of the crude enzyme solution in mL. V represents the volume of crude enzyme solution in mL. “t” represents the reaction time expressed in minutes.

The determination of protease activity was performed using a modified method as described in reference (Ramkumar et al., 2018). Casein was dissolved in a phosphoric acid buffer solution at pH 7.5 to produce a 2% casein solution as the reaction substrate. A mixture of 1.0 mL crude enzyme solution and 1.0 mL casein solution was incubated in a water bath at 40°C for 20 min, followed by the addition of 2.0 mL of 0.4 mol/L trichloroacetic acid solution. The mixture was then centrifuged at 8000 rpm for 10 minutes at 4°C. After discarding the supernatant, 1.0 mL of the resulting supernatant was added to a new test tube. After adding 5.0 mL of 0.4 mol/L Na_2_CO_3_ and 1.0 mL of folin phenol reagent to the reaction mixture, the solution was cooled to room temperature after a water bath at 40°C for 20 min. A blank control tube containing 1.0 mL of crude enzyme solution was boiled for 10 minutes to inactivate it. Then, 2.0 mL of 0.4 mol/L trichloroacetic acid solution and 1.0 mL of casein solution were added to both the measuring tube and the blank control tube, following the same procedure as before. The absorbance of the resulting solution at the wavelength of 660 nm was measured using an enzymoscope, and a linear equation was used to calculate the L-tyrosine standard curve. The mass of L-tyrosine in the measuring and blank control tubes was recorded as B1 and B0, respectively. Under standard assay conditions, the amount of enzyme required to release 1 μmol of tyrosine per minute from hydrolyzing casein is considered as the unit of protease activity (U). The formula for calculating protease activity is as follows:


(2) $$\text{X}=\frac{\left(B\times \text{V}\times n\right)}{\left(t\times V^{\prime }\right)}$$

In equation (2), X represents protease activity, expressed in U/g. B represents the stands for L-tyrosine production (B1-B0) in μg. V is the volume of the reaction solution in mL. “n” represents the dilution ratio of the crude enzyme solution. “t” is the reaction time, expressed in min. V’ is the volume of the crude enzyme solution in mL.

The amylase activity was determined using the improved method as described in reference (Yao et al., 2019). To perform the assay, 1.0 mL of crude enzyme solution and 1.0 mL of a 2% soluble starch solution were combined and mixed. After a 30-min water bath at 60°C, 2.0 mL of DNS solution was added. The mixture was then subjected to a boiling water bath for 10 min and cooled to a constant volume of 25 mL with distilled water. A blank control tube containing 1.0 mL of crude enzyme solution was boiled for 10 min to inactivate the enzyme. The remaining steps were the same as those used in the determination tube. The absorbance of the 520 nm wavelength was measured using an enzyme standard instrument, and the results were used to calculate the maltose standard curve equation. The mass of maltose in the measuring and blank control tubes was recorded as C1 and C0, respectively. A unit of amylase activity (U) was defined as the amount of enzyme required to hydrolyze soluble starch per minute to release 1 μmol maltose under standard assay conditions. The activity of amylase was calculated using the following formula:


(3) $$\text{X}=\frac{\left(C\times 1000\times n\right)}{\left(V\times \text{t}\times 342\right)}$$

In equation (3), X represents the activity of amylase, measured in U/g. C represents the yield of Maltose (C1-C0), measured in mg. 1000 is the conversion factor, with 1 mmol equivalent to 1000 μmol. “n” represents the dilution ratio of the crude enzyme solution. V is the volume of crude enzyme solution, measured in mL. “t” represents the reaction time, measured in minutes. Finally, 342 is relative molecular weight of maltose.

The lipase activity was determined using an improved method as described in reference (Joseph et al., 2012). To perform the assay, 100 μL p-nitrophenol palmitate solution (10 mmol/L), 700 μL of Tris HCl (pH 8.8) buffer solution, and 200 μL of crude enzyme solution were added to a centrifuge tube in sequence and mixed. After a 20-min incubation at 37°C water bath, 1.0 mL of 0.5 mol/L trichloroacetic acid solution was added and mixed well before standing for 5 min to complete the reaction. Next, 3 mL of 0.5 mol/L NaOH solution was added to mix well. As a blank control, 200 μL of distilled water was used instead of the crude enzyme solution, and the remaining steps were the same as those used in the determination tube. The absorbance of the 410 nm wavelength was measured using an enzyme standard instrument, and the results were used to calculated the standard cure equation for p-Nitrophenol. The mass of p-Nitrophenol in the measuring and blank control tubes was recorded as D1and D0, respectively. A unit of lipase activity (U) was defined as the amount of enzyme required to release 1 mol of p-nitrophenol from the p-nitrophenol palmitate per minute under standard assay conditions. The activity of lipase was calculated using the following formula:


(4) $$\text{X}=\frac{\left(D\times \text{V}\times n\right)}{\left(t\times V^{\prime }\right)}$$

In equation (4), X represents the lipase activity, measured in U/g. D represents the generation amount of p-nitrophenol (D1-D0), measured in μmol. V denotes the final volume of the reaction solution, measured in mL. “n” is the dilution ratio of the crude enzyme solution. “t” represents the reaction time, measured in min. V’ represents the volume of crude enzyme solution, measured in mL.

### 2.8 16S rRNA gene sequencing of cecal contents

The cecal contents were analyzed for total bacterial DNA using the Magnetic Soil and Stool DNA Kit (DP712). The purity and concentration of the extracted DNA were determined by Agilent 5400 agarose gel electrophoresis. Primers 515F (5′-GTGCCAGCMGCCGCGGTAA-3′) and 806R (5′-GGACTACHVGGGTWTCTAAT-3′) were used to amplify V4 region of the 16S rRNA gene in the bacterial DNA. The amplified products were detected using 2% agarose gel electrophoresis. DNA sequencing library was constructed with DNA library preparation kit and quantified using Qubit and qPCR. Finally, the Novaseq6000 system from Beijing Novogene Bioinformatics Technology Co., Ltd., was used for sequencing.

The original sequencing data was first filtered, followed by OTU clustering/denoising and species classification analysis to generate a species abundance spectrum for OTU and other classification levels. The OTU species abundance spectrum after data homogenization was then analyzed for OTU abundance and diversity index, as well as the statistical analysis of species annotation and community structure at different classification levels. R software’s pheatmap package was used to draw a correlation heat map between cecal digestive enzyme activity and flora, in order to identify microbial flora or species with strong correlation with cecal digestive enzyme activity.

### 2.9 Statistical analysis

The results were presented as mean ± SD and were evaluated using a student’s *t*-test. The statistical analysis showed that *P* < 0.05 was considered significant, while *P* < 0.01 was considered extremely significant. The phenotypic data was presented using GraphPad Prism (version 9.0, GraphPad Software Inc, San Diego, CA, USA).

## 3 Results

### 3.1 Complete genome information of *Bacillus velezensis* TS5

**FIGURE 1:**
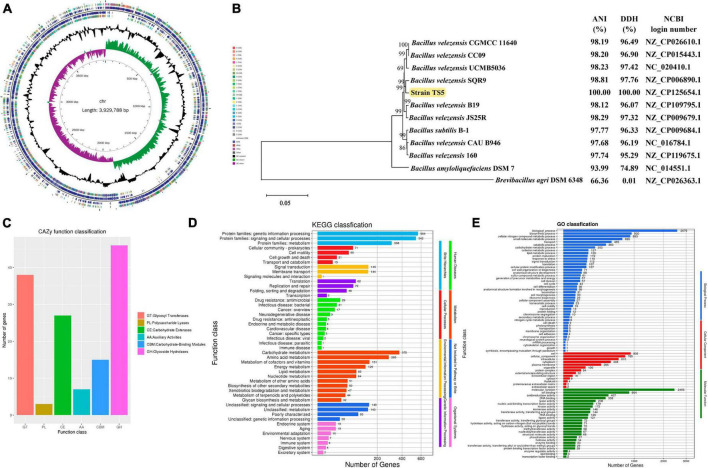
Genome analysis results of *Bacillus velezensis* TS5. **(A)** Genomic map of *B. velezensis* TS5. **(B)** Phylogenetic tree of *B. velezensis* TS5 based on genomic sequence. **(C)** Functional annotation of CAZy databases of *B. velezensis* TS5. **(D)** Functional annotation of KEGG databases of *B. velezensis* TS5. **(E)** Functional annotation of GO databases of *B. velezensis* TS5.

The genome of *Bacillus velezensis* TS5 is a circular chromosome with a total length of 3, 929, 788 bp and an average GC-content of 46.50% (Table 1). It contains 3873 protein-coding sequences, accounting for 88.59% of the total length, along with 82 ncRNAs, 86 tRNAs, and 27 rRNAs (Table 1). And its genome lacks CRISPRs. From inside to outside, the diagrammatic of the genome circle shows that the first circle represents scale, the second circle represents GC Skew, the third circle represents GC-content, the fourth and seventh circles represent COG to which each CDS belongs, the fifth and sixth circles represent the positions of CDS, tRNA, and rRNA on the genome (Figure 1A). Phylogenetic analysis based on multi-site sequence fragment analysis revealed strain TS5 and standard strain *Bacillus velezensis* SQR9 (NCBI login number: NZ_CP006890.1) to be part of the same branch (Figure 1B). The DDH and ANI homology indices were calculated between strain TS5 and 11 standard strains, and the results showed that strain TS5 had the highest homology with standard strain *Bacillus velezensis* SQR9 (DDH = 97.76%, ANI = 98.81%). These values meet the requirements for the same species of microorganisms, with a DDH greater than 95% and an ANI greater than 70%.

**TABLE 1:** Genome assembly and annotation of *Bacillus velezensis* TS5.

Types	Numbers	Genome percentage
Genome length	3929788 bp	/
Chromosome	1	/
Plasmid	0	/
GC-content	/	46.50%
Total number of protein coding genes	3873	/
The total length of protein coding genes	3481215 bp	88.59%
5S rRNA	9	0.03 %
16S rRNA	9	0.35 %
23S rRNA	9	0.67 %
tRNA	86	0.17 %
ncRNA	82	0.32 %
CRISPRs	0	/

The genome of *Bacillus velezensis* TS5 has been annotated with 136 genes in the carbohydrate active enzymes database (CAZy) (Figure 1C). The data revealed that 46 genes related to glycoside hydrolase (GHs) accounted for 33.82%, while 38 genes related to glycosyl transferases (GTs), represented 27.94%. Additionally, 27 genes related to carbohydrate esterases (CEs) made up 19.85%, and 15 genes related to carbohydrate binding modules (CBMs), accounted for 11.03%. Seven genes related to auxiliary activities (AAs) were found, representing 5.15%, while three genes related to polysaccharide lyases (PLs), accounted for 2.21%. These findings suggest that the metabolic activity of *Bacillus velezensis* TS5 is primarily focused on the breakdown of food substances.

*Bacillus velezensis* TS5 was found to contain 14 cellulase genes, including Beta-glucosidase, Exo-beta-1,4-glucanase, Glucan 1,3-beta-glucosidase, Glucan 1,4-beta-glucosidase, Exo-1,3-1,4-glucanase, Endo-1,3(4)-beta-glucanase, and Endoglucanase. These proteins encoded by cellulase genes belong the GH1, GH3, GH16, GH30, and GH51 families (Table 2). In addition, 43 hemicellulase genes were identified, such as Alpha-L-arabinofuranosidase, Xylan 1,4-beta-xylosidase, Alpha-galactosidase, Alpha-glucuronidase, Xylanase, Xyloglucanase, Beta-mannanase, Beta-1,3-xylanase, Beta-xylosidase, Endo-beta-1,6-galactanase, Endo-beta-1,4-galactanase, Alpha-1,6-mannanase, and Acetyl xylan esterase. The proteins encoded by these genes belong to the GH3, GH4, GH11, GH16, GH26, GH30, GH43, GH51, GH53, GH76, CE1, CE3, CE4, CE6, CE7, and CE12 families (Table 2). The results of the annotation suggest that *Bacillus velezensis* TS5 has the potential to degrade both cellulose and hemicellulose. This finding is significant for applications in biotechnology and industrial processes that require efficient degradation of plant cell wall polysaccharides.

**TABLE 2:** Cellulase and hemicellulase genes in the genome of *Bacillus velezensis* TS5.

Classification	CAZy	Count	Enzyme classification	EC numbers	Gene ID^a^
Cellulose- related	GH1	3	Beta-glucosidase	EC 3.2.1.21	gene1168 gene1935 gene3634
	GH1	3	Exo-beta-1,4-glucanase	EC 3.2.1.74	gene1168 gene1935 gene3634
	GH3	1	Beta-glucosidase	EC 3.2.1.21	gene184
	GH3	1	Glucan 1,3-beta-glucosidase	EC 3.2.1.58	gene184
	GH3	1	Glucan 1,4-beta-glucosidase	EC 3.2.1.74	gene184
	GH3	1	Exo-1,3-1,4-glucanase	EC 3.2.1.-	gene184
	GH16	1	Endo-1,3(4)-beta-glucanase	EC 3.2.1.6	gene3677
	GH30	1	Beta-glucosidase	EC 3.2.1.21	gene1935
	GH51	2	Endoglucanase	EC 3.2.1.4	gene2588 gene2610
Hemicellulose-related	GH3	1	Alpha-L-arabinofuranosidase	EC 3.2.1.55	gene184
	GH3	1	Xylan 1,4-beta-xylosidase	EC 3.2.1.37	gene184
	GH4	4	Alpha-galactosidase	EC 3.2.1.22	gene2762 gene2097 gene791 gene3619
	GH4	4	Alpha-glucuronidase	EC 3.2.1.139	gene2097 gene2762 gene3619 gene791
	GH11	1	Xylanase	EC 3.2.1.8	gene3426
	GH16	1	Xyloglucanase	EC 3.2.1.151	gene3677
	GH26	1	Beta-mannanase	EC 3.2.1.78	gene3638
	GH26	1	Beta-1,3-xylanase	EC 3.2.1.32	gene3638
	GH30	1	Beta-xylosidase	EC 3.2.1.37	gene1935
	GH30	1	Endo-beta-1,6-galactanase	EC:3.2.1.164	gene1935
	GH43	1	Beta-xylosidase	EC 3.2.1.37	gene1812
	GH43	1	Beta-1,3-xylosidase	EC 3.2.1.-	gene1812
	GH43	1	Alpha-L-arabinofuranosidase	EC 3.2.1.55	gene1812
	GH43	1	Xylanase	EC 3.2.1.8	gene1812
	GH51	2	Alpha-L-arabinofuranosidase	EC 3.2.1.55	gene2588 gene2610
	GH53	1	Endo-beta-1,4-galactanase	EC 3.2.1.89	gene1162
	GH76	1	Alpha-1,6-mannanase	EC 3.2.1.101	gene3847
	CE1	6	Acetyl xylan esterase	EC 3.1.1.72	gene1064 gene1144 gene123 gene1931 gene2924 gene825
	CE3	2	Acetyl xylan esterase	EC 3.1.1.72	gene1987 gene406
	CE4	7	Acetyl xylan esterase	EC 3.1.1.72	gene770 gene932 gene1437 gene1641 gene165 gene2407 gene3657
	CE6	1	Acetyl xylan esterase	EC 3.1.1.72	gene2425
	CE7	1	Acetyl xylan esterase	EC 3.1.1.72	gene314
	CE12	2	Acetyl xylan esterase	EC 3.1.1.72	gene2406 gene2712

Gene ID^a^: In this study, a total of 3873 genes were obtained in the genome of *Bacillus velezensis* TS5. Each gene was assigned a unique identification number, ranged from “Gene 1” to “Gene 3873”, according to its position within the genome sequence.

In total, 2,163 KEGG annotation genes were identified in the genome of *Bacillus velezensis* TS5 (Figure 1D). These genes were assigned to six different signaling pathway levels. The distributing of these genes among various categories was as follows: 1333 genes related to metabolism, 290 genes related to environmental information processing, 207 genes related to genetic information processing, 160 genes related to cellular processes, 96 genes related to human diseases, and 67 genes related to biological system. Among the metabolism-related genes, the main annotations included carbohydrate metabolism (375), amino acid metabolism (285), metabolism of cofactors and vitamins (151), energy metabolism (129), lipid metabolism (85), nucleotide metabolism (84), metabolism of other amino acids (51), biosynthesis of other secondary metabolites (50), xenobiotics biodegradation and metabolism (47), metabolism of terpenoids and polyketides (44) and glycine biosynthesis and metabolism (32). These findings suggest that *Bacillus velezensis* TS5 has strong abilities in carbohydrates and proteins metabolism, which could be valuable for applications in various industries such as food production and biotechnology.

*Bacillus velezensis* TS5 was analyzed for functional prediction using the GO database, and the genic functions were divided into three categories: biological processes, cellular components, and molecular functions (Figure 1E). The majority of genes were assigned to various biological processes, including biosynthesis (2579), biosynthetic process (930), cellular nitrogen compound metabolic process (893), small molecule metabolic process (696), transport (465), catabolic process (363), carbohydrate metabolic process (202), cofactor metabolic process (127), lipid metabolic process (126), protein maturation (119), response to stress (116), signal transduction (112), translation (107), and cellular protein modification process (107). In addition, *Bacillus velezensis* TS5 was found to have a diverse range of cellular components and molecular functions. In terms of cellular components, most genes were associated with cell (905), cellular component (765), intracellular (610), cytoplasm (448), plasma membrane (286), and organelle (100). This suggests that *Bacillus velezensis* TS5 has the potential to perform various cellular processes within its cells. In terms of molecular function, the majority of genes were associated with molecular function (2456), ion binding (864), oxidoreductase activity (407), DNA binding (358), lyase activity (208), nucleic acid binding transcription factor activity (179), kinase activity (172), isomerase activity (146), transferase activity and transferring acyl groups (144), RNA binding (126), and ligase activity (121). These findings suggest that *Bacillus velezensis* TS5 has the ability to perform a wide range of molecular functions within its cells.

The genome of *Bacillus velezensis* TS5 was searched for multiple gene clusters related to the synthesis of antibacterial metabolites using the antiSMASH tool (Table 3). The identified gene clusters include Butirosin, Macroactin, Bacillaene, Difficidin, Bacilliactin, and Bacilysin, which have known antibacterial abilities. Additionally, Fengytin has been previously identified as having antifungal abilities, while Surfactin has various potentials such as antibacterial, antifungal, and antiviral properties. These findings suggest that *Bacillus velezensis* TS5 may have a diverse range of natural products with antimicrobial activities.

**TABLE 3:** Secondary metabolites gene clusters determined by antiSMASH analysis in *Bacillus velezensis* TS5 genome.

Cluster	Type	Most similar known cluster	Length (bp)	Function	Similarity (%)
1	NRPS	Surfactin	63977	Multiple	82
2	PKS-like	Butirosin	41244	Antibacterial	7
3	Terpene	/	17408	/	/
4	Lanthipeptide class II	/	28888	/	/
5	TransAT-PKS	Macrolactin H	87835	Antibacterial	100
6	TransAT-PKS	Bacillaene	100565	Antibacterial	100
7	NRPS	Fengycin	134310	Antifungal	100
8	Terpene	/	21883	/	/
9	T3PKS	/	41100	/	/
10	TransAT-PKS	Difficidin	93792	Antibacterial	100
11	NRPS	Bacillibactin	51791	Antibacterial	100
12	other	Bacilysin	41418	Antibacterial	100

Based on gene annotation results, the genome of *Bacillus velezensis* TS5 identified numerous genes that function as probiotic markers, such as acid tolerance, bile salt tolerance, adhesion, antioxidant, and digestive enzyme (Supplementary Tables 1, 2). These probiotic genes are an important prerequisite for *Bacillus velezensis* TS5 to exert probiotic functions. In addition, compared with the CARD database, the results showed that the genome alignment of *Bacillus velezensis* TS5 identified 40 genes related to antibiotic resistance, 22 genes related to antibiotic target, and 3 genes related to antibiotic biosynthesis (Supplementary Table 3). However, there were no acquired resistance genes found in the genome of *Bacillus velezensis* TS5 by using the ResFinder tool to compare the resistance genes. Comparing the genome sequence of *Bacillus velezensis* TS5 with the VFDB database, only a few virulence genes related to motility, adhesion, stress survival, immune modulation, and nutritional/metabolic factor were detected in the genome data of *Bacillus velezensis* TS5 (Supplementary Table 4). However, there were no virulence genes found in the genome of *Bacillus velezensis* TS5 using the Virulence finder tool.

### 3.2 Results on the probiotic functions of *Bacillus velezensis* TS5 in mice

**FIGURE 2:**
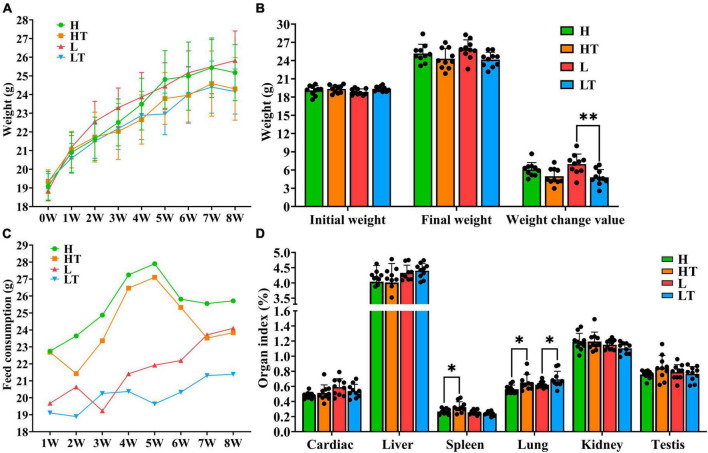
Mouse growth indicators. **(A,B)** Body weight changes in mice. **(C)** Consumption of mice feed. **(D)** Organ index of mice. “*” indicates a significant difference (*P* < 0.05), “**” indicates a significant difference (*P* < 0.01).

The results of the study showed that mice in the probiotic group (HT and LT groups) had slower weight growth compared to the control group (H and L groups), as depicted in Figures 2A, B. Additionally, mice on the high fiber diet (H and HT groups) consumed more feed than those on the low fiber diet (L and LT groups), while the probiotic group (HT and LT groups) had lower feed consumption than the control group (H and L groups) (Figure 2C). The organ index results for mice were presented in Figure 2D. The spleen index of the HT group was significantly higher than that of the H group (*P* < 0.05), but there was no significant difference in other organ indices (heart, liver, lungs, kidneys, and testes) (*P* > 0.05). There was also no significant difference in all organ indices (heart, liver, spleen, lungs, kidneys, and testes) between the LT group and L group (*P* > 0.05).

The blood routine results of the mice are presented in Table 4, and it can be seen that the control group (H and L groups) had normal blood routine indicators. In contrast, the hemoglobin content in the blood of the HT group mice was found to be significantly higher than that of the H group (*P* < 0.05), while the hemoglobin content in the blood of the LT group mice was also significantly higher than that of the L group (*P* < 0.05). The other blood routine indicators of the probiotic group (HT and LT groups) were within the normal range, with only slightly increases in hemoglobin content compared to the reference value. This increase may be attributed to a reduction in water consumption by the mice, leading to an increase in blood concentration.

**TABLE 4:** Blood routine results of mice.

Parameter	H group	HT group	L group	LT group	Reference value
Leukocyte (10^9^/L)	6.08 ± 2.42	6.46 ± 2.21	7.01 ± 2.77	9.38 ± 1.92	0.80-10.60
Neutrophils (10^9^/L)	0.74 ± 0.28^b^	0.90 ± 0.26^b^	0.89 ± 0.43^b^	1.32 ± 0.27^a^	0.23-3.60
Lymphocyte (10^9^/L)	4.94 ± 1.99	5.21 ± 1.99	6.04 ± 2.31	8.20 ± 1.77	0.60-8.90
Monocyte (10^9^/L)	0.21 ± 0.10	0.14 ± 0.05	0.18 ± 0.11	0.24 ± 0.11	0.04-1.40
Erythrocyte (10^12^/L)	9.98 ± 1.38	10.65 ± 0.69	9.54 ± 1.11	10.36 ± 0.85	6.50-11.50
Hemoglobin (g/L)	162.75 ± 19.85^ab^	174.33 ± 9.71^a^	156.38 ± 18.81^b^	170 ± 13.84^a^	110-165
Platelet (10^9^/L)	1017.22 ± 278.09	1151.91 ± 217.84	868.33 ± 350.26	1023 ± 133.88	400-1600

Peer data shows significant differences using different letters (*P* < 0.05), while the same or no letters indicate no significant differences (*P* > 0.05).

**FIGURE 3:**
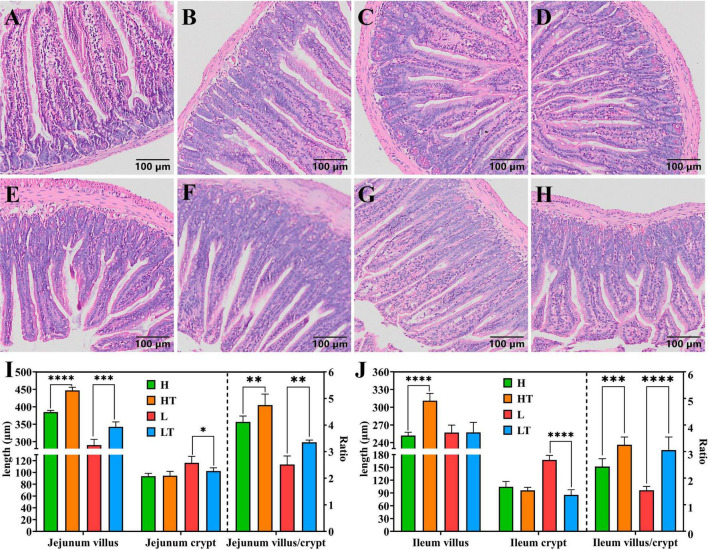
HE staining results of mice jejunum and ileum. **(A–D)** Pathological section results of the jejunum in groups H, HT, L, and LT (100×). **(E–H)** Pathological section results of the ileum in groups H, HT, L, and LT (100×). **(I)** The villus length, crypt depth and the ratio of villus length to crypt depth in jejunum. **(J)** The villus length, crypt depth and the ratio of villus length to crypt depth in ileum. “*” indicates a significant difference (*P* < 0.05), “**” indicates a significant difference (*P* < 0.01), “***” indicates a significant difference (*P* < 0.001), “****” indicates a significant difference (*P* < 0.0001).

The tissues of the jejunum and ileum from all mice were found to be intact, with clear structure and no obvious pathological changes (Figures 3A–H). The results of measuring the length of intestinal villi and the depth of crypts (Figures 3I, J) revealed that the height of jejunal and ileal villi in the HT group were significantly longer than that in the H group (*P* < 0.0001), while the depth of jejunal and ileal crypts in the LT group was significantly lower than that in the L group (*P* < 0.05). Additionally, the ratio of villi length to crypt depth in the HT group was significantly higher than that in the H group (*P* < 0.01), while it was also significantly higher in the LT group compared to the L group (*P* < 0.01). These findings suggest that *Bacillus velezensis* TS5 not only did not cause any damage to the jejunum and ileum of mice, but also improved their intestinal morphology by increasing villus length and decreasing crypt depth.

**FIGURE 4:**
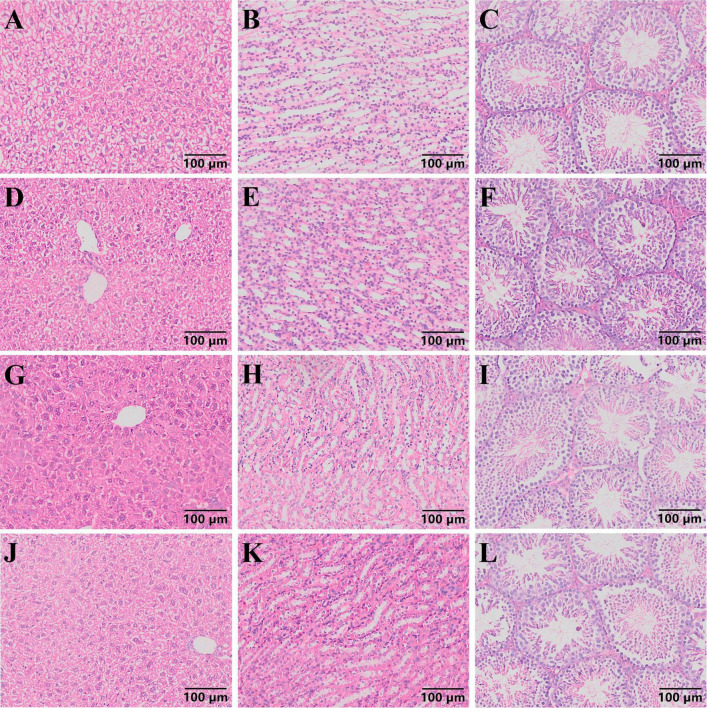
Pathological section results of mouse liver, kidney and testis (100 ×). **(A–C)** Pathological sections of liver, kidney, and testicles in group H mice. **(D–F)** Pathological sections of liver, kidney, and testicles in group HT mice. **(G–I)** Pathological sections of liver, kidney, and testicles in group L mice. **(J–L)** Pathological sections of liver, kidney, and testicles in group LT mice.

The pathological section results of mouse liver, kidney, and testes (Figure 4) revealed that in each group of mice, the central vein and portal area could be seen in the liver under microscope. The structure of lobules of liver was normal, with no swelling of necrosis of hepatocytes observed, and they were arranged in strips. No abnormality was found in the kidney, and the capillaries of the renal corpuscle in the renal cortex showed no abnormal changes. The morphology of the tubular wall cells was normal, and the boundaries of the renal vesicles were clear. The results of testicular sections indicated that the size of seminiferous tubules in each group of mice was essentially the same, and all levels of spermatogenic cells in the tubules were arranged neatly. Sperm were present in the tubules, and the interstitial tissue was normal without congestion or bleeding, indicating a normal tissue morphology and structure.

**FIGURE 5:**
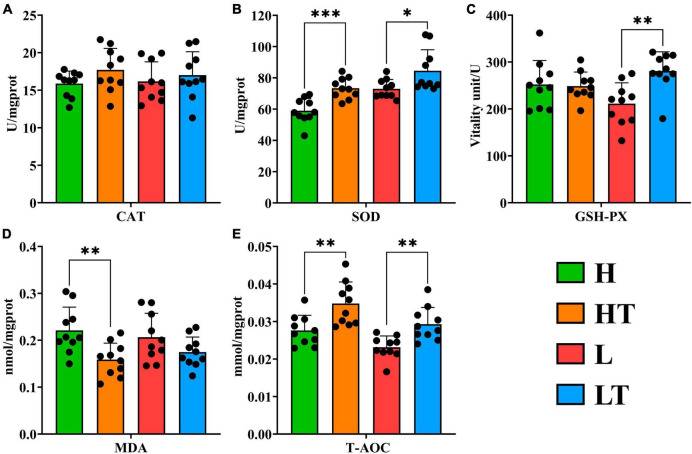
Antioxidant test results of liver in mice. **(A)** CAT in mouse liver. **(B)** SOD in mouse liver. **(C)** GSH-PX in mouse liver. **(D)** MDA in mouse liver. **(E)** T-AOC in mouse liver. “*” indicates a significant difference (*P* < 0.05), “**” indicates a significant difference (*P* < 0.01), “***” indicates a significant difference (*P* < 0.001).

The results of the anti-oxidation analysis of mouse liver showed that the Catalase (CAT) activity in the probiotic groups (HT and LT groups) was higher than that in their respective control groups (L and LT groups), but the difference was not significant (*P* > 0.05) (Figure 5A). In addition, the activities of Superoxide dismutase (SOD) in the HT group were significantly higher than those in the H group (*P* < 0.001), and the activities of SOD in the LT group were significantly higher than those in the L group (*P* < 0.05) (Figure 5B). Furthermore, the activities of Glutathione peroxidase (GSH Px) in the LT group were significantly higher than those in the L group (*P* < 0.01) (Figure 5C). The content of Malondialdehyde (MDA) in the HT group was also lower than that in the H group (*P* < 0.01) (Figure 5D). Finally, the total antioxidant capacity (T-AOC) in the probiotic group (HT and LT groups) was significantly higher than that in their respective control groups (L and LT groups) (*P* < 0.01) (Figure 5E). These findings suggest that *Bacillus velezensis* TS5 enhances the antioxidant capacity of the mouse liver and has a positive impact on the immune system to a certain extent.

**FIGURE 6:**
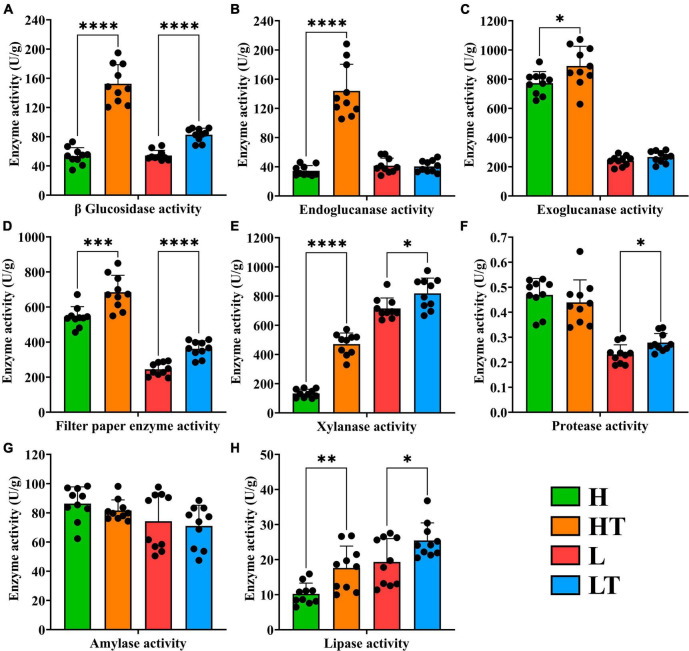
Digestive enzyme activity of cecum contents in mice. **(A)** β glucosidase activity. **(B)** Endoglucanase activity. **(C)** Exoglucanase activity. **(D)** Filter paper enzyme activity. **(E)** Xylanase activity. **(F)** Protease activity. **(G)** Amylase activity. **(H)** Lipase activity. “*” indicates a significant difference (*P* < 0.05), “**” indicates a significant difference (*P* < 0.01), “***” indicates a significant difference (*P* < 0.001), “****” indicates a significant difference (*P* < 0.0001).

The results of the enzyme activity in mice cecum contents are shown in Figure 6. The activities of β glucosidase (*P* < 0.0001), endoglucanase (*P* < 0.0001), exoglucanase (*P* < 0.05), filter paper enzyme (*P* < 0.001), xylanase (*P* < 0.0001) and lipase activity (*P* < 0.01) in the HT group were significantly higher than those in the H group. Similarly, the activities of β glucosidase (*P* < 0.0001), filter paper enzyme (*P* < 0.0001), xylanase (*P* < 0.05), protease (*P* < 0.05) and lipase (*P* < 0.05) in the LT group were significantly higher than those in the L group. These findings indicated that *Bacillus velezensis* TS5 can enhance the activities of β glucosidase, filter paper enzyme, xylanase and lipase in the mice cecum, thereby facilitating intestinal nutrient digestion.

**FIGURE 7:**
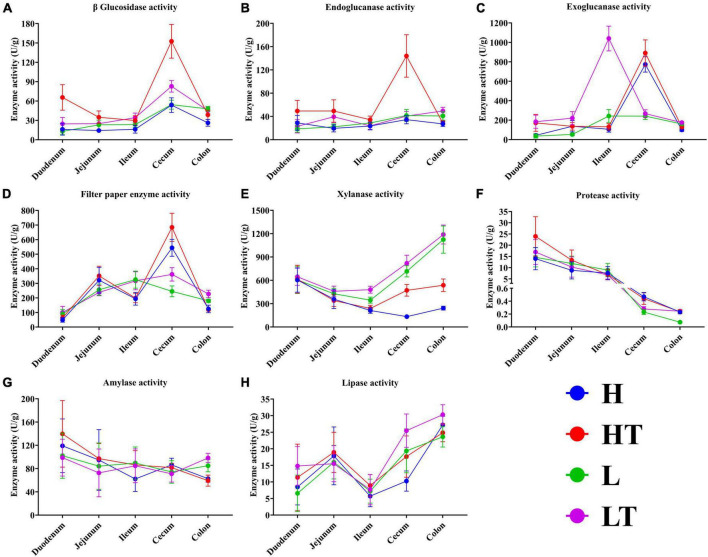
Digestive enzyme activities of whole intestinal contents in mice. **(A)** β glucosidase activity. **(B)** Endoglucanase activity. **(C)** Exoglucanase activity. **(D)** Filter paper enzyme activity. **(E)** Xylanase activity. **(F)** Protease activity. **(G)** Amylase activity. **(H)** Lipase activity.

The results of the digestive enzyme activity in mice’s whole intestinal contents are depicted in Figure 7. The cecum exhibited higher activities of cellulase activities (including β glucosidase, endoglucanase, exoglucanase, and filter paper enzyme activities) compared to other intestinal segments. Additionally, the colon had relatively high activities of xylanase and lipase. It is noteworthy that the activities of protease and amylase gradually decreased from the foregut to the hindgut, with the highest levels observed in the duodenum. These findings suggest that *Bacillus velezensis* TS5 has the ability to increase digestive enzyme activity throughout the entire intestinal tract of mice, particularly in the cecum, colon, and duodenum.

**FIGURE 8:**
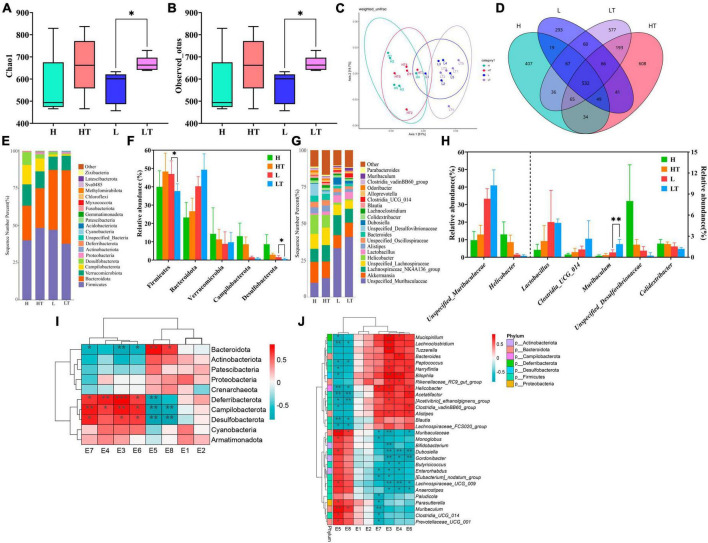
Results and analysis of 16S rRNA amplification and sequencing in the mice cecum. **(A)** Box plot of Chao1’s diversity index. **(B)** Box plot of observed OTU’s diversity index. **(C)** Principal co-ordinates analysis (PCoA) with weighted unifrac of microbiota. **(D)** Venn diagram of the cecal microbiota. **(E)** Histogram of the top 20 phylum level microbiota in relative abundance. **(F)** Histogram of relative abundance of dominant bacterial phyla in the cecum. **(G)** Histogram of the top 20 genus level microbiota in relative abundance. **(H)** Histogram of relative abundance of dominant bacterial genera in the cecum. **(I)** Thermogram of correlation between cecal digestive enzyme activity and phylum level microbiota. **(J)** Thermogram of correlation between cecal digestive enzyme activity and genus level microbiota. E1-E8 represents the activities of β-glucosidase, endoglucanase, exoglucanase, filter paper enzyme, xylanase, protease, amylase, and lipase, respectively. “*” indicates a significant difference (*P* < 0.05), “**” indicates a significant difference (*P* < 0.01).

The Chao1 index results of Alpha diversity (Figure 8A) showed that the species richness of cecal bacteria in the probiotic group (HT and LT groups) was higher than that of the respective control groups (H and L groups), but the difference was not statistically significant (*P* > 0.05). The observed OTU index (Figure 8B) also indicated that the microbial diversity in the cecum of the HT group mice was higher than that of the H group mice (*P* > 0.05), while the microbial diversity in the cecum of the LT group mice was lower than that of the L group mice (*P* > 0.05). The principal coordinate analysis (PCoA) results of beta diversity showed (Figure 8C) that the samples of the high fiber diet group (H and HT groups) and the low fiber diet group (L and LT groups) were gathered together and separated from each other, indicating that there was a significant difference in species diversity between the two groups. The petal plot (Figure 8D) further revealed that there were 532 common OTUs in all four groups, with the number of unique OTUs in the cecal contents of mice in the H, HT, L, and LT groups being 407, 608, 293, and 574, respectively. Notably, the number of OTUs in the cecum of mice in the probiotic group (HT and LT groups) was higher than that of the corresponding control groups (H and L groups), indicating that *Bacillus velezensis* TS5 had a positive impact on increasing the diversity of the cecal microbiota in mice.

The results of the phylum level analysis (Figures 8E, F) revealed that Firmicutes, Bacteroidota, Verrucomicrobiota, Campilobacterota, and Desulfobacteria were the most dominant phyla in the cecum of all four groups of mice. Comparing the H group with the HT group, the abundance of Verrucomicrobiota, Campylobacter and Desulfobacteria was lower in the HT group, while the abundance of Firmicutes and Bacteroidota was higher. Conversely, when comparing the L group with the LT group, there was a decrease in the abundance of Firmicutes, Campylobacter, and Desulfobacteria, and an increase in the abundance of Bacteroidota and Verrucomicrobiota in the LT group. At the genus level (Figures 8G, H), the comparison between the H group and the HT group showed an increase in the abundance of *unspecified_Muribaaculaceae*, *Lactobacillus*, *Clostridia_UCG_014*, and *Muribaculum*, while a decrease in the abundance of *Helicobacter*, *Unspecified_Desulfovibrionacea, and Colidextribacter*. In contrast, when comparing the L group with the LT group, there was an increase in the abundance of *Unspecified_Muribaculaceae, Clostridia_UCG_014*, and *Muribaculum*, while a decreased in the abundance of *Helicobacter*, *Unspecified_ Desulfovibrionaceae, and Colidextribacter* in the LT group.

The correlation analysis between the phylum level microbiota and digestive enzyme activities was presented in Figure 8I. The results showed that the activities of exoglucanase (E3), filter paper enzyme (E4), protease (E6), and amylase (E7) were positively correlated with the abundances of Deferribactere, Campilobacterota, and Desulfobacterota, but negatively correlated with Bacteroidota. On the other hand, the activities of xylanase (E5) and lipase (E8) were positively correlated with Bacteroidota, and negatively correlated with the abundance of Deferribactere, Campilobacterota, and Desulfobacterota. The correlation analysis between the genus level microbiota and digestive enzyme activities was shown in Figure 8J. The results indicated that the activity of exoglucanase (E3) was positively correlated with the abundance of *Mucispirillum*, *Lachnoclostridium*, *Tuzzerella*, and *Bilophila*, while negatively correlated with the abundance of *Muribaculaceae*, *Bifidobacterium*, *Dubosiella*, *Gordonibacter*, and *Lachnospiraceae_UCG_009*. The activity of filter paper enzyme (E4) was positively correlated with the abundance of Bilophila, and significantly negatively correlated with the abundance of *Dubosiella* and *Lachnospiraceae_UCG_009*. The activity of protease (E6) was negatively correlated with *Dubosiella* and *Gordonibacter*. Additionally, the activity of xylanase (E5) was positively correlated with the abundance of *Muribaculaceae* and *Muribaculum*, while negatively correlated with the abundance of *Helicobacter* and *Acetatifactor*. Interestingly, the bacterial microbiota that were positively correlated with the activities of exoglucanase, filter paper enzyme, protease, and amylase were negatively correlated with the activities of xylanase and lipase, and vice versa, respectively.

## 4 Discussion

### 4.1 Complete genome sequencing analysis provides probiotic potential for *Bacillus velezensis* TS5

Genomic analysis can reveal the presence of genes encoding probiotic properties such as acid and bile tolerance, epithelial adhesion, and production of antibacterial substances (Kapse et al., 2019; Pereira et al., 2019; Soni et al., 2021). The genome sequence analysis of *Bacillus velezensis* TS5 also identified several stress-resistant genes including *DnaK*, *OppA*, *Eno*, *nhaC*, *nhaX*, and *nhaK* (Supplementary Table 1). These findings are consistent with previous studies which have reported the presence of bile salt tolerance genes (*DnaK*, *OppA*, and *Eno*) in probiotic *Bacillus velezensis* FS26 (Sam-On et al., 2023). The Na/H antiporter (*nhaC*, *nhaX*) and universal stress proteins (*nhaK*) play a crucial role in Na resistance and pH homeostasis, allowing the bacteria to survive in acidic conditions (Fujisawa et al., 2005). Moreover, our results indicate that *B. velezensis* TS5 is capable of producing antimicrobial compounds and exhibiting broad-spectrum antimicrobial activity.

The genome of *Bacillus velezensis* TS5 contains three genes, lipoprotein signal peptidase (*LspA* gene), glutamine binding periplasmic protein (*GlnH* gene), and elongation factor Tu (*Tuf* gene), which are also known as mucus adhesion domain protein (MucBPs) (Supplementary Table 1). These proteins play a grucial role in host adhesion, automatic aggregation, and/or coaggregation with pathogenic bacteria (Sam-On et al., 2023). The findings are consistent with previous studies which have reported the presence of similar MucBPs (*LspA*, *GlnH*, and *Tuf* genes) in probiotic *Bacillus velezensis* FS26 (Sam-On et al., 2023).

Probiotics have been widely acknowledged for their ability to enhance the host’s antioxidant capacity by activating antioxidant related pathways or increasing the activity of antioxidant enzymes (Xu et al., 2019; Zhao et al., 2021). For instance, glutathione peroxidase (*bsaA*) is known to protect cells from oxidative stress by reducing hydrogen peroxide to water (Lubos et al., 2011). Previous studies have also reported the presence of multiple genes encoding catalase and peroxidase in the genome of *Bacillus subtilis* DE111, a safe commercial probiotic (Mazhar et al., 2022). In this study, we found that *Bacillus velezensis* TS5 contains multiple genes encoding both glutathione peroxidase, catalases, peroxidases, and thioredoxin which further validated its antioxidant properties (Supplementary Table 1).

The process of decomposing carbohydrates such as cellulose, proteoglycans, and starch into mono or oligo saccharides that can be absorbed by intestinal epithelium requires the essential CAZymes (Shang et al., 2022). The GH family enzymes play a crucial role in this process by hydrolyzing glycosidic bonds (Roth et al., 2017). In *Bacillus velezensis* TS5, 11 GH family members were identified, including GH1, GH3, GH16, GH30, and GH51 that encoded cellulase, while GH1 and GH3 were primarily β-glucosidases (Suzuki et al., 2013). These enzymes coordinately degrade cellulose, while other GH family members could also degrade hemicellulose. For instance, GH3, GH4, GH11, GH16, GH26, GH30, GH43, GH51, GH53, and GH76 could degrade hemicellulose. Among them, GH26 was mainly composed of β-1,4 mannanases that could hydrolyze mannan, galactomannan, and glucomannan (Taylor et al., 2005). Additionally, GH43 was an important member of xylan degradation (Zanphorlin et al., 2019), while GH51 could dissociate arabinogalactan and arabinomannan in cell walls promoting the degradation of pectin (Im et al., 2012). GH53 had been reported as an endo-1,4-β-galactanase and had the ability to hydrolyze 1,4-β-D-galactoside bonds (Sakamoto et al., 2013). In addition, carbohydrate esterases related to xylan decomposition were identified in *Bacillus velezensis* TS5, including CE1, CE3, CE4, CE6, CE7, and CE12. Among these, CE3, CE4, and CE7 were acetyl xylan esterases that could promote the dissolution of xylan (Zhang et al., 2011). The abundance of cellulase and hemicellulase genes further indicated that *Bacillus velezensis* TS5 has the potential to degrade both cellulose and hemicellulose. These findings suggest that *Bacillus velezensis* TS5 is a promising candidate for the degradation of complex carbohydrates in various industrial applications.

Proteases, amylases, and lipases are enzymes that can decompose proteins, carbohydrates, and lipids into amino acids, aldehydes, amines, free fatty acids, organic acids, and esters (Heo et al., 2021). *Bacillus velezensis* TS5 was found to possess 21 protease or peptidase genes (Supplementary Table 2), which suggest that it has the ability to degrade proteins. In addition, *Bacillus velezensis* TS5 also encoded seven α-amylase gene and five lipases and esterases genes (Supplementary Table 2). These findings suggest that *Bacillus velezensis* TS5 has the potential to promote nutrient digestion. A study indicated that the cellulase and phytase secreted by *Bacillus velezensis* LB-Y-1 contribute to the release of nutrients in Arbor Acres broiler chickens feed (Li et al., 2023). The protease and amylase secreted by *Bacillus velezensis* LB-Y-1 enhanced the intestinal digestibility of broilers, thereby promoting nutrient digestion.

Bacterial resistance can be divided into two categories: one is inherent resistance that develops during the formation of microorganisms and usually does not transfer, and the other is acquired resistance with high levels of transfer potential (Holzapfel et al., 2018). The acquired resistance genes in probiotic strains are considered a major threat to the spread of drug resistance between different bacterial species (Fu et al., 2022). There were no acquired resistance genes found in the genome of *Bacillus velezensis* TS5, indicated a lower risk of spreading resistance. Although the analysis of the *Bacillus velezensis* TS5 genome against the VFDB revealed the presence of some putative virulence genes, they could not be considered really harmful. Genes encoding hemolysin A (hlyA), cytolysin (cyl), enterotoxins haemolysin BL (Hbl), non-hemolytic enterotoxin (Nhe), and cytotoxin K (CytK), which are well-known potential virulence factors, were missing in *Bacillus velezensis* TS5 (Schoeni and Wong, 2005; Dietrich et al., 2021). In addition, no truly toxic toxin coding genes were found in the genome of *Bacillus velezensis* TS5. More importantly, we have demonstrated the safety of *Bacillus velezensis* TS5 through mice experiments.

### 4.2 The probiotic function in mice is an important prerequisite for *Bacillus velezensis* TS5 to become a potential digestive probiotic

Recently, high-fiber feeds such as wheat bran, rapeseed meal, and cottonseed meal have been increasingly utilized in monogastric animal feed production (Shang et al., 2022). However, these high fiber contents present a challenge for monogastric animals to digest, resulting in the wasteful consumption of limited feed resources. A high-fiber diet can lead to an increase in chyme emptying rate, which residence time of chyme in the gastrointestinal tract, decreased the contact between digestive enzymes and chyme, and ultimately lowers the digestibility of nutrients (Jha and Leterme, 2012). Therefore, enhancing the degradation of fiber components in feed is an effective approach to improve feed digestibility. Previous studies have demonstrated that incroporating probiotics, such as *Bacillus*, into animal feed can promote digestion and absorption, thereby enhancing feed utilization and production performance (Manhar et al., 2016; Mahoney et al., 2020; Khalid et al., 2021). Soni et al. (2021) supplemented *Bacillus velezensis* ZBG17 to the broiler diet, resulting in a significantly improvement in the feed utilization rate and humoral immunity response of broilers. Genomic analysis of this bacterium revealed that it lacked genes related to safety hazards, demonstrating its potential as a direct feeding microorganism in the poultry industry. Li et al. (2020) isolated a strain of *Bacillus velezensis* JT3-1 with potent antibacterial activity from yak feces, which improve the growth performance of calves and alleviated calf diarrhea to some extent. Although numerous studies suggested that *Bacillus velezensis* is a promising probiotic, its probiotic properties still require further exploration. Therefore, in this study, we established two distinct cellulose diets for mice and administered gavage *Bacillus velezensis* TS5 for an entire 8-week period, with the aim of investigating the *in vivo* probiotic properties of *Bacillus velezensis* TS5.

Probiotics are live microorganisms that have beneficial effects on the host by colonizing the gut and modifying the composition of certain gut microbiota (Gupta and Garg, 2009). A previous study examined the probiotic potential of *Bacillus licheniformis* (named D1 and D2) and *Bacillus pumilus* (named X1 and X2) isolated from yak intestines, and the results showed that supplementation with these probiotics increased daily weight gain and reduced feed conversion rate in mice (Zeng et al., 2022). Yang et al. (2023) reported that *Bacillus subtilis* BS-Z15 can regulate the gut microbiota of mice through its metabolites to reduce weight gain. In this study, we found that *Bacillus velezensis* TS5 slowed down the weight gain of mice, which was not affected by the cellulose content in the feed (high fiber or low fiber feed). Moreover, *Bacillus velezensis* TS5 increased the diversity and richness of the cecal microbiota in mice and altered the content of certain microbiota (increased the content of Bacteroides and reduced the content of Campylobacter and Desulfobacteria). Previous research has shown that the main function of the Bacteroidetes phylum is to degrade carbohydrates and proteins and participate in lipid metabolism (Yang et al., 2023). At the genus level, *Bacillus velezensis* TS5 increased the abundance of *Lactobacillus* in the mice’s cecum, which is consistent with the findings reported by Donaldson et al. (2016) and Yang et al. (2023) that there is an association between increased abundance of beneficial gut bacteria, such as *Lactobacillus* and *Bifidobacterium* and weight loss. This indicated that *Bacillus velezensis* TS5 regulated weight gain in mice by altering the intestinal bacterial structure, which could potentially prevent obesity in human.

Previous research has demonstrated that supplementing probiotics helps maintain the balance of gut microbiota in the host, while also increasing the number of beneficial bacteria and reducing the colonization of harmful bacteria in the intestinal tract (Baldwin et al., 2018; Li et al., 2019a; Fuerniss et al., 2022). The cecum, a significant fermentation site in mice, contains a rich microbial community. Previous studies have shown that *Bacillus velezensis* isolated from Tibetan yaks can enhance the gut microbiota of mice (Li et al., 2019c). In this study, oral administration of *Bacillus velezensis* TS5 decreased the relative abundance of potential harmful bacteria such as *Helicobacter*, *Unspecified_Desulfovibrionaceae*, and *Colidextribacter* in the cecum and improved the composition of the mice’s cecum microbiota to a certain extent.

The small intestine is a vital part of the animal body for absorption, and research has shown that probiotic supplementation can enhance this process by increasing the length of intestinal villus and reducing the depth of crypts. In this study, the pathological sections showed that the jejunum and ileum tissues in mice appeared healthy with no apparent lesions (Figure 3). By evaluating the villus length and crypt depth of these regions, it was discovered that the V/C values of the probiotic group were significantly higher than those of the control group (*P* < 0.01). This demonstrates that *Bacillus velezensis* TS5 promotes nutrient absorption and improves intestinal morphology in mice.

Previous research has reported that probiotics can enhance antioxidant capacity by increasing the activity of antioxidant enzymes in the host body (Wang et al., 2017). Li et al. (2019b) found that *Bacillus velezensis* BV2 could increase SOD and GSH-PX activity in the liver of mice, reduce MDA content, and increase T-AOC, thereby improving the antioxidant capacity of mice. In this study, the results showed that compared to the control group, the probiotic group increased the activity of CAT, SOD, and GSH-Px in the mouse liver, decreased the MDA content, and increased T-AOC. This suggests that *Bacillus velezensis* TS5 has the potential to improve the antioxidant capacity of mice.

During intestinal colonization, probiotics can metabolize and produce extracellular digestive enzyme such as protease, lipase, and cellulase, which can further improve the digestive capacity of the host. *Bacillus* may be the most important source of proteases due to their ability to produce a large number of neutral and alkaline proteolytic enzymes with significant characteristics, such as high stability under extreme temperatures, pH, organic solvents, detergents, and oxidizing compounds (Contesini et al., 2018). Li et al. (2019b) found that *Bacillus velezensis* BV2 enhanced the growth of small intestine (duodenum, jejunum, and ileum) in mice by increasing α-amylase, lipase, and trypsin activities. Jang et al. (2023) reported that supplementing red sea bream (Pagrus major) with *Bacillus sp.* PM8313 significantly increased trypsin and lipase activity in the anterior midgut. In this study, we measured the activity of digestive enzymes in the intact intestinal contents of mice. Our results showed that *Bacillus velezensis* TS5 promoted nutrient digestion by increasing the activities of β glucosidase, filter paper enzyme, xylanase and lipase in the mice’s cecum (Figure 6). Furthermore, the activity of other intestinal contents was also increased to varying degrees (Supplementary Figures 1–4).

## 5 Conclusion

In summary, the genomic and *in vivo* studies of *Bacillus velezensis* TS5 isolated from Tibetan sheep provided valuable insights into its efficacy as a probiotic in mice study. The genome mining of *Bacillus velezensis* TS5 identified numerous genes that function as probiotic markers in terms of host intestinal adhesion, antioxidant, production of antibacterial substances and digestive enzyme, as well as acid and bile salt tolerance. Animal experiments demonstrated that *Bacillus velezensis* TS5 can improve the activity of intestinal digestive enzymes and liver antioxidant capacity in mice, as well as the morphology of small intestine and cecum microbiota structure. These findings provided a theoretical basis for the clinical application and development of *Bacillus velezensis* TS5 as a new feed additive.

## Data availability statement

The raw data of the bacterial complete genome and the 16S rRNA sequencing data are deposited in the NCBI repository, with the accession numbers CP125654.1 and PRJNA1028384, respectively.

## Ethics statement

The animal study was approved by the Animal Ethical and Welfare Committee of Sichuan Agricultural University [SYXK (Chuan) 2019-187]. The study was conducted in accordance with the local legislation and institutional requirements.

## Author contributions

BC: Data curation, Methodology, Validation, Visualization, Writing – original draft, Software. YiZ: Software, Visualization, Writing – original draft. LD: Data curation, Investigation, Writing – original draft. XG: Data curation, Investigation, Software, Writing – original draft. XL: Investigation, Validation, Writing – original draft. KP: Methodology, Software, Writing – review and editing. DZ: Writing – review and editing, Project administration, Supervision. XN: Writing – review and editing, Conceptualization, Methodology. YaZ: Funding acquisition, Methodology, Project administration, Supervision, Writing – review and editing.
