# Supplementary material for: Complete genome analysis of Bacillus velezensis TS5 and its potential as a probiotic strain in mice
Source: Front Microbiol. 2023 Dec 6;14:1322910. doi: 10.3389/fmicb.2023.1322910 (PMC10731255; doi:10.3389/fmicb.2023.1322910)
Supplement: Supplementary file 1 [file Data_Sheet_1.docx]

**Complete genome analysis of *Bacillus velezensis* TS5 and its potential as a probiotic strain in mice**

**Benhao Chen^1,2†^, Yi Zhou^1,2†^, Lixiao Duan^1,2†^, Xuemei Gong^1,2^, Xingmei Liu^1,2^, Kangcheng Pan^1,2^, Dong Zeng^1,2*^, Xueqin Ni^1,2*^, Yan Zeng^1,2*^**

^1^Animal Microecology Institute, College of Veterinary Medicine, Sichuan Agricultural University, Chengdu, China

^2^ Engineering Research Center of Southwest Animal Disease Prevention and Control Technology, Ministry of Education of the People’s Republic of China, Chengdu, China

*Correspondence:

Yan Zeng

yanzeng@sicau.edu.cn

Tel: +8618583296107

Xueqin Ni

[xueqinni@foxmail.com](mailto:xueqinni@foxmail.com)

Dong Zeng

[zend@sicau.edu.cn](mailto:zend@sicau.edu.cn)

^†^ These authors contributed equally to this work and share first authorship.

Supplementary Material

## Supplementary tables

Table S1 List of putative probiotic genes of *Bacillus velezensis* TS5

| **Functional category** | **Gene symbol** | **Description/Function** | **Gene ID** |
| --- | --- | --- | --- |
| Acid tolerance | *atpA* | ATP synthase subunit alpha | gene3449 |
|  | *atpB* |  | gene3453 |
|  | *atpC* | ATP synthase epsilon chain | gene3446 |
|  | *atpD* | ATP synthase subunit beta | gene3447 |
|  | *atpG* | ATP synthase gamma chain | gene3448 |
|  | *cspB* | Cold shock protein | gene880 |
|  | *cspC* |  | gene501 |
|  | *cspD* |  | gene2005 |
|  | *yjbQ* | Sodium hydrogen exchanger | gene1116 |
|  | *atpF* | ATP synthase subunit b | gene3451 |
|  | *nhaC* | Na H antiporter | gene533 |
|  | *nhaK* |  | gene3086 |
|  | *mleN* |  | gene2170 |
|  | *nhaC* |  | gene2325 |
|  | *mrpA* | Na H antiporter subunit | gene2885 |
|  | *mrpB* |  | gene2886 |
|  | *mrpD* |  | gene2888 |
|  | *mrpE* |  | gene2889 |
|  | *mrpG* |  | gene2891 |
|  | *ldh* | L-lactate dehydrogenase | gene301 |
|  | *mdh* | Malate dehydrogenase | gene2650 |
|  | *ald* | Alanine dehydrogenase | gene2916 |
|  | *gabD* | Succinate-semialdehyde dehydrogenase | gene386 |
|  | *yjgC* | Probable oxidoreductase | gene1177 |
|  | *yoaE* |  | gene1870 |
|  | *yrhE* | Putative formate dehydrogenase | gene2458 |
|  | *fdhD* | Sulfur carrier protein | gene3436 |
|  | *clpC* | ATP-dependent CLP protease ATP-binding subunit | gene96 |
|  | *clpE* |  | gene1334 |
|  | *yjbM* | GTP pyrophosphokinase | gene1112 |
|  | *relA* |  | gene2498 |
|  | *ywaC* |  | gene3611 |
|  | *pyk* | Pyruvate kinase | gene2661 |
|  | *pgi* | Phosphohexose isomerase | gene2859 |

Table S1 List of putative probiotic genes of *Bacillus velezensis* TS5 (continued 1)

| **Functional category** | **Gene symbol** | **Description/Function** | **Gene ID** |
| --- | --- | --- | --- |
| Bile tolerance | *mreB* | Cell shape-determining protein | gene2538 |
|  | *mbl* |  | gene3407 |
|  | *oppA* | Oligopeptide-binding protein | gene1093 |
|  | *eno* | Enolase OS | gene3157 |
|  | *dnaK* | Chaperone protein | gene2381 |
|  | *dnaJ* |  | gene2380 |
|  | *yxeI* | Choloylglycine hydrolase | gene3640 |
|  | *nagB* | Glucosamine-6-phosphate deaminase | gene3262 |
|  | *pyrG* | CTP synthase | gene3480 |
| General stress resistance | *nhaX* | Universal stress protein | gene936 |
|  | *yxiE* |  | gene3683 |
| Antioxidant | *catD* | Putative oxidoreductase | gene798 |
|  | *catE* | Catechol-2,3-dioxygenase | gene799 |
|  | *ygaF* | Peroxiredoxin | gene838 |
|  | *ydfQ* | Thioredoxin | gene259 |
|  | *ydbP* |  | gene462 |
|  | *stoA* |  | gene1350 |
|  | *yneN* |  | gene1779 |
|  | *trxA* |  | gene2587 |
|  | *ytpP* |  | gene2735 |
|  | *yusE* |  | gene3009 |
|  | *trxB* | Thioredoxin reductase | gene3242 |
|  | *ahpF* | Alkyl hydroperoxide reductase | gene3755 |
|  | *RBAM_003480* | NADP reductase | gene320 |
|  | *RBAM_029160* |  | gene2935 |
|  | *namA* | NADH flavin oxidoreductase | gene2214 |
|  | *yqiG* |  | gene2257 |
|  | *ydbD* | Catalase | gene450 |
|  | *cotJC* |  | gene687 |
|  | *katA* |  | gene854 |
|  | *katX* |  | gene3639 |
|  | *katE* |  | gene3675 |
|  | *bsaA* | Glutathione peroxidase | gene2002 |
| Adhesion | *lspA* | Lipoprotein signal peptidase | gene1513 |
|  | *tuf* | Elongation factor Tu | gene122 |
|  | *slp* | Pal-related lipoprotein | gene1436 |
|  | *ykuI* | EAL-domain containing protein | gene1375 |
|  | *hag* | Flagellin | gene3294 |
|  | *epsD* | Putative glycosyltransferase | gene3199 |
|  | *epsL* | sugar transferase | gene3191 |

Table S1 List of putative probiotic genes of *Bacillus velezensis* TS5 (continued 2)

| **Functional category** | **Gene symbol** | **Description/Function** | **Gene ID** |
| --- | --- | --- | --- |
| Adhesion | *epsM* | Sugar o-acyltransferase | gene3190 |
|  | *epsN* | Putative pyridoxal phosphate-dependent aminotransferase | gene3189 |
|  | *srtD* | Sortase D | gene889 |
|  | *glnH* | ABC transporter glutamine-binding protein | gene2480 |

Table S2 Potential enzyme genes identified in *Bacillus velezensis* TS5 genome

| **Subsystem** | **Functional category** | **Gene symbol** | **Description/Function** | **Gene ID** |
| --- | --- | --- | --- | --- |
| Proteases |  | *prsA* | peptidylprolyl cis-trans isomerase | gene963 |
|  |  | *clpP* | ATP-dependent Clp protease | gene3219 |
|  |  | *clpC* | ATP-dependent CLP protease ATP-binding subunit | gene96 |
|  | Peptidase-S9 | *prsW* | Involved in the degradation of specific anti-sigma factors | gene2109 |
|  | Serine protease | *htrB* | Serine protease | gene3036 |
|  |  | *aprX* |  | gene1696 |
|  | Metallopeptidases | *htpX* | Protease HtpX homolog | gene1313 |
| Peptide metabolism | Oligopeptide transporters | *oppA* | Oligopeptide ABC transporter | gene1093 |
|  |  | *oppB* | Oligopeptide transport system permease protein | gene1094 |
|  |  | *oppC* |  | gene1095 |
|  |  | *oppD* | Oligopeptide ABC transporter | gene1096 |
|  |  | *oppF* |  | gene1097 |
|  | Dipeptide transporters | *dppA* | D-aminopeptidase | gene1262 |
|  |  | *dppB* | Binding-protein-dependent transport systems | gene1263 |
|  |  | *dppC* |  | gene1264 |
|  |  | *dppD* | ABC transporter | gene1265 |
|  |  | *dppE* | Dipeptide-binding protein | gene1266 |
|  | Aminopeptidases | *pepA* | Probable cytosol aminopeptidase | gene2928 |
|  |  | *map* | Methionine aminopeptidase | gene145 |
|  | Endopeptidase | *pepF* | Oligoendopeptidase f | gene3033 |
|  | Tripeptidase | *pepT* | Cleaves the N-terminal amino acid of tripeptides | gene3663 |
| Amylase | Alpha amylase | *ycdG* | Alpha amylase | gene281 |
|  |  | *amyE* |  | gene299 |
|  |  | *amyE* |  | gene300 |
|  |  | *bbmA* |  | gene3227 |
|  |  | *treA* |  | gene748 |
|  |  | *yugT* |  | gene2851 |
|  |  | *malL* |  | gene3221 |

Table S2 Potential enzyme genes identified in *Bacillus velezensis* TS5 genome (continued 1)

| **Subsystem** | **Functional category** | **Gene symbol** | **Description/Function** | **Gene ID** |
| --- | --- | --- | --- | --- |
| Lipases and Esterases | Esterase | *estA* | Triacylglycerol lipase | gene271 |
|  | Lipases | *mhqD* | Phospholipase Carboxylesterase | gene1931 |
|  |  | *est* | Carboxylesterase | gene3122 |
|  |  | *pnbA* |  | gene3204 |
|  |  | *ytpA* | Phospholipase | gene2788 |

Table S3 Antimicrobial resistance genes detected in the genome of *Bacillus velezensis* TS5

| **Gene ID** | **Antibiotic type** | **Resistance**  **gene** | **Function** | **Source organism** | **Identity**  **(%)** |
| --- | --- | --- | --- | --- | --- |
| gene6 | Antibiotic target | *gyrB* | DNA gyrase subunit B | *Bacillus amyloliquefaciens subsp. plantarum str.* FZB42 | 99.69 |
| gene7 | Antibiotic target | *gyrA* | DNA gyrase subunit A | *Bacillus amyloliquefaciens subsp. plantarum str.* FZB42 | 99.62 |
| gene116 | Antibiotic target | *rpoB* | DNA-directed RNA polymerase subunit beta | *Bacillus amyloliquefaciens subsp. plantarum str.* FZB42 | 100.00 |
| gene117 | Antibiotic target | *rpoC* | DNA-directed RNA polymerase subunit beta | *Bacillus amyloliquefaciens subsp. plantarum str.* FZB42 | 99.83 |
| gene121 | Antibiotic target | *fusA* | elongation factor G | *Bacillus amyloliquefaciens subsp. plantarum str.* FZB42 | 100.00 |
| gene463 | Antibiotic target | *ddl* | D-alanyl-alanine synthetase A | *Bacillus amyloliquefaciens subsp. plantarum str.* FZB42 | 99.15 |
| gene472 | Antibiotic target | *alr* | hypothetical protein | *Bacillus amyloliquefaciens subsp. plantarum str.* FZB42 | 98.46 |
| gene962 | Antibiotic target | *GBAA_1020* | conserved hypothetical protein | *Bacillus anthracis str.* Ames Ancestor | 46.27 |
| gene1405 | Antibiotic target | *BBR47_36110* | signal peptidase I | *Brevibacillus brevis* NBRC 100599 | 46.45 |
| gene1448 | Antibiotic target | *SaurJH1_1191* | GTP-binding protein TypA | *Staphylococcus aureus subsp. aureus* JH1 | 77.15 |
| gene1505 | Antibiotic target | *SaurJH1_1272* | alanine racemase domain-containing protein | *Staphylococcus aureus subsp. aureus* JH1 | 55.86 |
| gene1626 | Antibiotic target | *dxr* | 1-deoxy-D-xylulose 5-phosphate reductoisomerase | *Bacillus amyloliquefaciens subsp. plantarum str.* FZB42 | 98.34 |
| gene1788 | Antibiotic target | *parE* | DNA topoisomerase IV subunit B | *Bacillus amyloliquefaciens subsp. plantarum str.* FZB42 | 99.69 |
| gene1789 | Antibiotic target | *parC* | DNA topoisomerase IV subunit A | *Bacillus amyloliquefaciens subsp. plantarum str.* FZB42 | 99.26 |

Table S3 Antimicrobial resistance genes detected in the genome of *Bacillus velezensis* TS5 (continued 1)

| **Gene ID** | **Antibiotic type** | **Resistance**  **gene** | **Function** | **Source organism** | **Identity**  **(%)** |
| --- | --- | --- | --- | --- | --- |
| gene1994 | Antibiotic target | *dfrA* | dihydrofolate reductase | *Bacillus subtilis subsp. subtilis str.* 168 | 75.16 |
| gene2144 | Antibiotic target | *BBR47_36110* | signal peptidase I | *Brevibacillus brevis* NBRC 100599 | 47.75 |
| gene2260 | Antibiotic target | *yncD* | hypothetical protein | *Bacillus amyloliquefaciens subsp. plantarum str.* FZB42 | 99.49 |
| gene2302 | Antibiotic target | *sipW* | signal peptidase I | *Bacillus anthracis str.* Ames Ancestor | 49.71 |
| gene2468 | Antibiotic target | *greA* | transcription elongation factor GreA | *Bacillus amyloliquefaciens subsp. plantarum str.* FZB42 | 100.00 |
| gene2531 | Antibiotic target | *rplU* | 50S ribosomal protein L21 | *Azoarcus sp.* BH72 | 48.54 |
| gene3188 | Antibiotic target | *epsO* | putative pyruvyl transferase | *Bacillus subtilis subsp. subtilis str.* 168 | 67.52 |
| gene3194 | Antibiotic target | *epsI* | putative polysaccharide pyruvyl transferase | *Bacillus subtilis subsp. subtilis str.* 168 | 75.98 |
| gene6 | Antibiotic resistance | *gyrB* | DNA gyrase subunit B | *Staphylococcus aureus subsp. aureus* MRSA252 | 70.03 |
| gene7 | Antibiotic resistance | *gyrA* | acetolactate synthase | *Staphylococcus aureus subsp. aureus* MRSA252 | 65.10 |
| gene83 | Antibiotic resistance | *folP* | dihyropteroate synthase | *Streptococcus pyogenes* MGAS8232 | 46.43 |
| gene116 | Antibiotic resistance | *rpoB* | DNA-directed RNA polymerase subunit beta | *Staphylococcus aureus subsp. aureus* MRSA252 | 80.73 |
| gene117 | Antibiotic resistance | *rpoC* | DNA-directed RNA polymerase subunit beta | *Staphylococcus aureus subsp. aureus* MRSA252 | 76.82 |
| gene119 | Antibiotic resistance | *StrA* | Unknown product | *Haemophilus influenzae* | 71.53 |
| gene122 | Antibiotic resistance | *tufA* | translational elongation factor TU | *Staphylococcus aureus subsp. aureus* Mu50 | 86.80 |
| gene269 | Antibiotic resistance | *lmrA* | transcriptional repressor of lmrAB and yxaGH operons | *Bacillus subtilis subsp. subtilis str.* 168 | 72.19 |
| gene310 | Antibiotic resistance | *tmrB* | ATP-binding tunicamycin resistance protein | *Bacillus subtilis subsp. subtilis str.* 168 | 76.65 |
| gene528 | Antibiotic resistance | *RBAM_005660* | chloramphenicol/florfenicol resistance protein | *Bacillus amyloliquefaciens subsp. plantarum str.* FZB42 | 99.43 |
| gene684 | Antibiotic resistance | *aadK* | aminoglycoside 6-adenylyltransferase | *Bacillus subtilis subsp. subtilis str.* 168 | 63.04 |

Table S3 Antimicrobial resistance genes detected in the genome of *Bacillus velezensis* TS5 (continued 2)

| **Gene ID** | **Antibiotic type** | **Resistance**  **gene** | **Function** | **Source organism** | **Identity**  **(%)** |
| --- | --- | --- | --- | --- | --- |
| gene771 | Antibiotic resistance | *bcrA* | Unknown product | *Bacillus licheniformis* | 79.08 |
| gene772 | Antibiotic resistance | *bcrB* | Unknown product | *Bacillus licheniformis* | 55.61 |
| gene773 | Antibiotic resistance | *bcrC* | Unknown product | *Bacillus licheniformis* | 57.07 |
| gene774 | Antibiotic resistance | *vanRF* | two-component response regulator | *Paenibacillus popilliae* ATCC 14706 | 48.71 |
| gene810 | Antibiotic resistance | *mprF* | phosphatidylglycerol lysyltransferase involved in lysinylation of phospholipids | *Bacillus subtilis subsp. subtilis str.* 168 | 78.31 |
| gene835 | Antibiotic resistance | *SAV1866* | ABC transporter homolog | *Staphylococcus aureus subsp. aureus* Mu50 | 61.25 |
| gene1022 | Antibiotic resistance | *blt* | efflux transporter | *Bacillus subtilis subsp. subtilis str.* 168 | 77.83 |
| gene1066 | Antibiotic resistance | *fosB* | Unknown product | *Staphylococcus epidermidis* | 61.59 |
| gene1138 | Antibiotic resistance | *AAA22562.1* | Unknown product | *Bacillus cereus* | 52.74 |
| gene1158 | Antibiotic resistance | *bla1* | beta-lactamase I | *Bacillus anthracis* | 64.05 |
| gene1275 | Antibiotic resistance | *ykkC* | efflux transporter | *Bacillus subtilis subsp. subtilis str.* 168 | 81.48 |
| gene1276 | Antibiotic resistance | *ykkD* | efflux transporter | *Bacillus subtilis subsp. subtilis str.* 168 | 81.73 |
| gene1416 | Antibiotic resistance | *adeC* | adenine deaminase | *Enterococcus faecium* DO | 47.13 |
| gene1663 | Antibiotic resistance | *pgsA* | phosphatidylglycerophosphate synthase | *Staphylococcus aureus subsp. aureus* Mu50 | 51.83 |
| gene1701 | Antibiotic resistance | *ABBFA_001145* | Quaternary ammonium compound-resistance protein qacE | *Acinetobacter baumannii* AB307-0294 | 47.52 |
| gene1733 | Antibiotic resistance | *AIA08936.1* | rifampin phosphotransferase | *Streptomyces sp.* WAC4747 | 55.50 |
| gene1788 | Antibiotic resistance | *grlB* | DNA topoisomerase IV subunit B | *Staphylococcus aureus subsp. aureus* MRSA252 | 71.19 |
| gene1789 | Antibiotic resistance | *grlA* | DNA topoisomerase IV subunit A | *Staphylococcus aureus subsp. aureus* MRSA252 | 57.29 |

Table S3 Antimicrobial resistance genes detected in the genome of *Bacillus velezensis* TS5 (continued 3)

| **Gene ID** | **Antibiotic type** | **Resistance**  **gene** | **Function** | **Source organism** | **Identity**  **(%)** |
| --- | --- | --- | --- | --- | --- |
| gene1994 | Antibiotic resistance | *dfrG* | dihydrofolate reductase | *Staphylococcus aureus* | 52.50 |
| gene1995 | Antibiotic resistance | *AAD01867.1* | thymidylate synthase | *Enterococcus faecalis* | 66.67 |
| gene2437 | Antibiotic resistance | *sat4* | streptothricine-acetyl-transferase | *Campylobacter coli* | 52.63 |
| gene2839 | Antibiotic resistance | *bacA* | undecaprenyl pyrophosphate phosphatase | *Escherichia coli str.* K-12 *substr.*MG1655 | 47.57 |
| gene3047 | Antibiotic resistance | *HMPREF*  *0351_10938* | LuxR family response regulator | *Enterococcus faecium* DO | 58.46 |
| gene3132 | Antibiotic resistance | *bcrA* | Unknown product | *Bacillus licheniformis* | 47.48 |
| gene3423 | Antibiotic resistance | *SAV2088* | cardiolipin synthetase | *Staphylococcus aureus subsp.aureus* Mu50 | 58.17 |
| gene3550 | Antibiotic resistance | *bcrA* | Unknown product | *Bacillus licheniformis* | 46.69 |
| gene3724 | Antibiotic resistance | *bcrA* | Unknown product | *Bacillus licheniformis* | 45.87 |
| gene3802 | Antibiotic resistance | *vanRM* | VanRM | *Enterococcus faecium* | 46.19 |
| gene427 | Antibiotic biosynthesis | *YBT020_12150* | pyruvate oxidase | *Bacillus thuringiensis serovar finitimus* YBT-020 | 54.59 |
| gene2567 | Antibiotic biosynthesis | *YBT020_07635* | acetolactate synthase catalytic subunit | *Bacillus thuringiensis serovar finitimus* YBT-020 | 66.67 |
| gene3361 | Antibiotic biosynthesis | *YBT020_04620* | acetolactate synthase | *Bacillus thuringiensis serovar finitimus* YBT-020 | 65.83 |

Table S4 Virulence genes detected in the genome of *Bacillus velezensis* TS5

| **Gene ID** | **Virulence factor classification** | **Virulence factor** | **Related gene** | **Virulence factor reference strain** |
| --- | --- | --- | --- | --- |
| gene1606 | Motility | Flagella | *fliP* | *Pseudomonas aeruginosa* PAO1 |
| gene122 | Adherence | EF-Tu | *tufA* | *Francisella tularensis subsp. tularensis* SCHU S4 |
| gene602 | Adherence | GroEL | *groEL* | *Clostridium difficile* 630 |
| gene96 | Stress survival | ClpC | *clpC* | *Listeria monocytogenes* EGD-e |
| gene1334 | Stress survival | ClpE | *clpE* | *Listeria monocytogenes* EGD-e |
| gene3219 | Stress survival | ClpP | *clpP* | *Listeria monocytogenes* EGD-e |
| gene3429 | Stress survival | Urease | *ureB* | *Helicobacter pylori* 26695 |
| gene1562 | Immune modulation | LPS | *acpXL* | *Brucella melitensis bv. 1 str.* 16M |
| gene2219 | Immune modulation | Capsule | *gndA* | *Klebsiella pneumoniae subsp. pneumoniae* NTUH-K2044 |
| gene3189 | Immune modulation | LPS | *LPG_RS03745* | *Legionella pneumophila subsp. pneumophila str. Philadelphia* 1 |
| gene3324 | Immune modulation | Capsule | *cps4I* | *Streptococcus pneumoniae* TIGR4 |
| gene3346 | Immune modulation | Capsule | *capC* | *Bacillus anthracis* |
| gene3347 | Immune modulation | Capsule | *capB* | *Bacillus anthracis* |
| gene3662 | Immune modulation | LOS | *galE* | *Haemophilus influenzae* Rd KW20 |
| gene3662 | Immune modulation | Capsule | *galE* | *Francisella tularensis subsp. tularensis* SCHU S4 |
| gene3662 | Immune modulation | Capsule | *galE* | *Acinetobacter baumannii* ACICU |
| gene992 | Nutritional/Metabolic factor | LplA1 | *lplA1* | *Listeria monocytogenes* EGD-e |
| gene2919 | Nutritional/Metabolic factor | Bacillibactin | *dhbF* | *Bacillus anthracis str. Sterne* |
| gene2921 | Nutritional/Metabolic factor | Bacillibactin | *dhbE* | *Bacillus anthracis str. Sterne* |
| gene2923 | Nutritional/Metabolic factor | Bacillibactin | *dhbA* | *Bacillus anthracis str. Sterne* |

## Supplementary figures


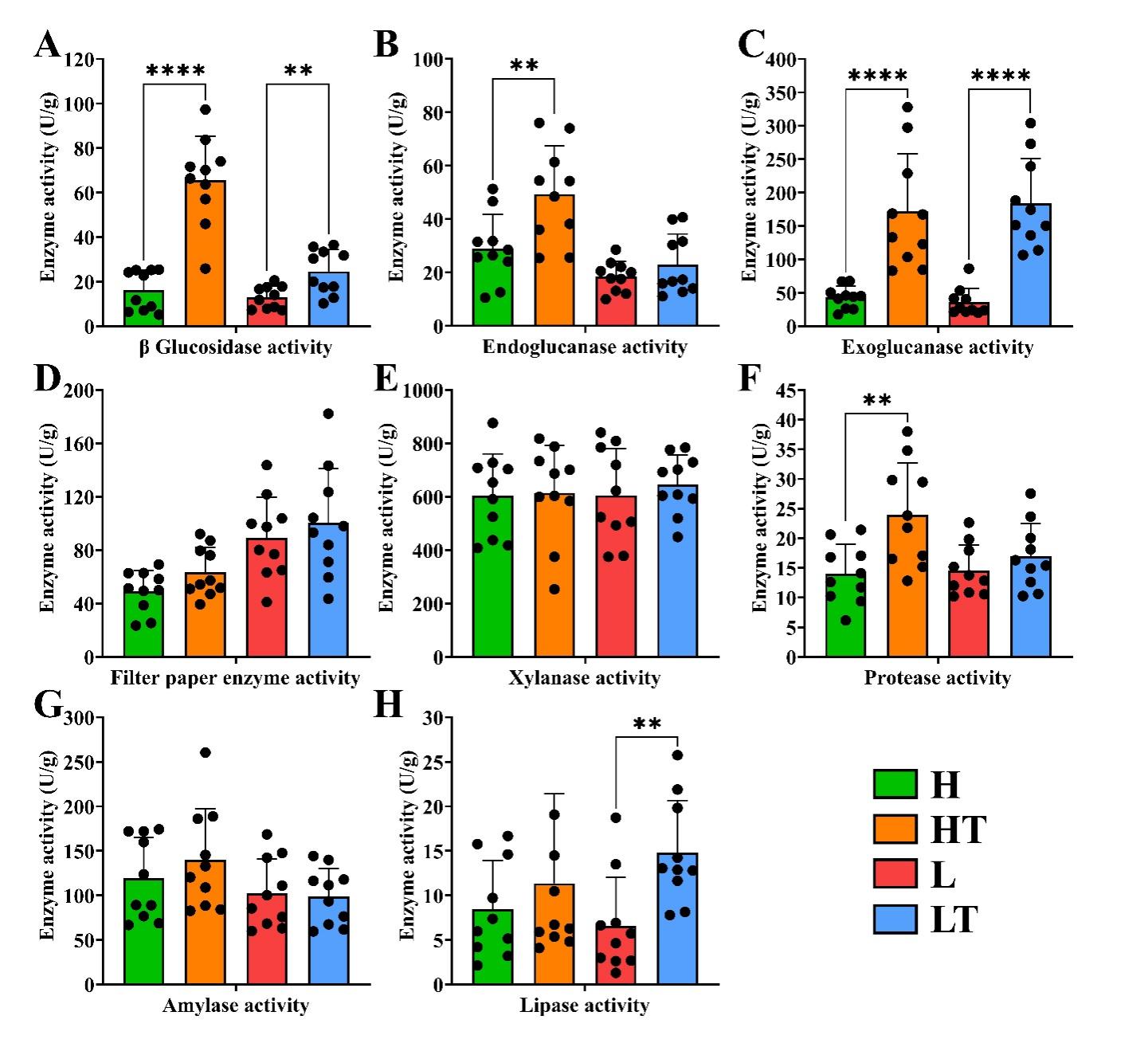


**Fig. S1 Digestive enzyme activity of duodenum contents in mice.** **(A)** β glucosidase activity. **(B)** Endoglucanase activity. **(C)** Exoglucanase activity. **(D)** Filter paper enzyme activity. **(E)** Xylanase activity. **(F)** Protease activity. **(G)** Amylase activity. **(H)** Lipase activity. “**” indicates a significant difference (*P* < 0.01), “****” indicates a significant difference (*P* < 0.0001).


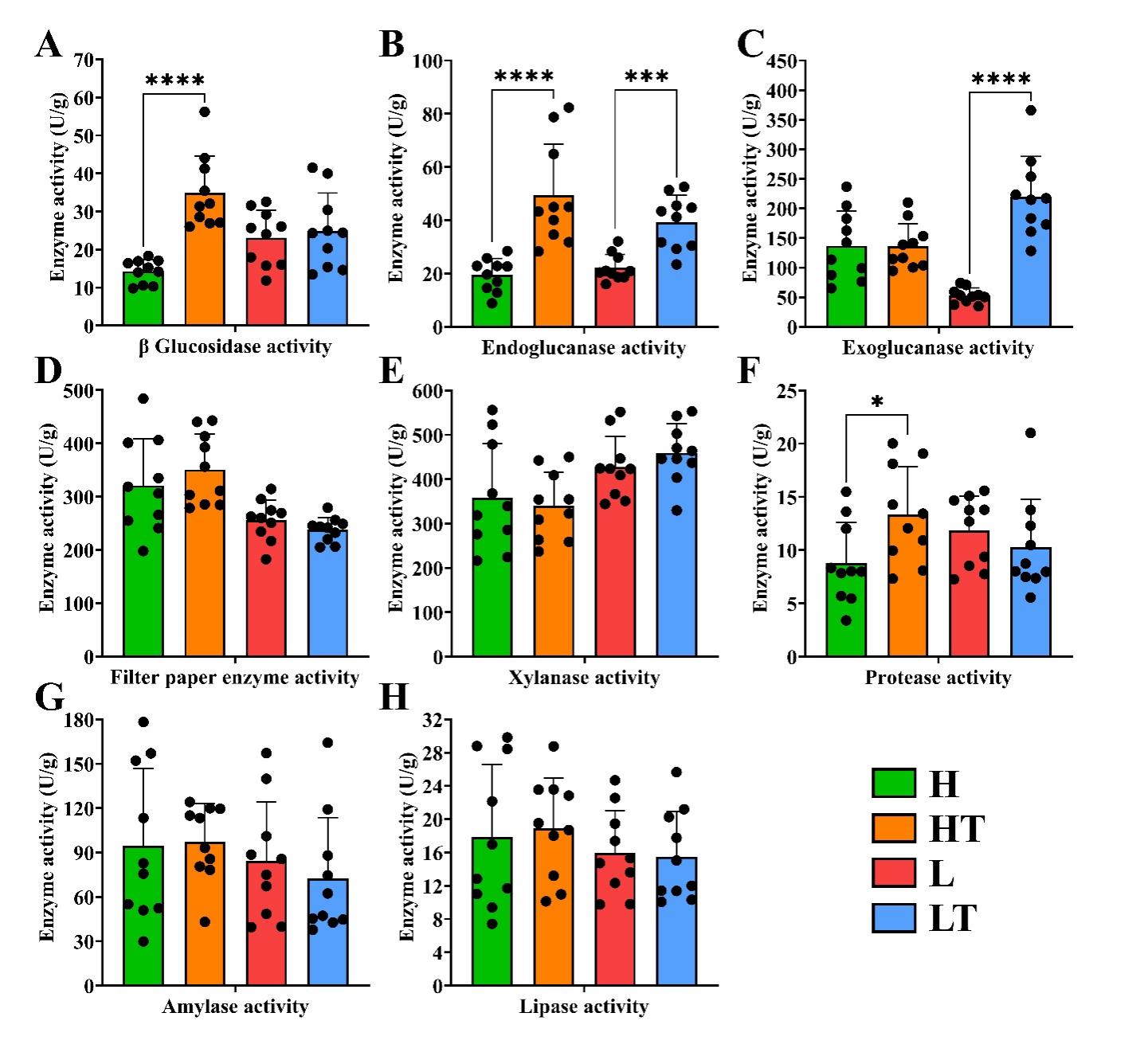


**Fig. S2 Digestive enzyme activity of jejunum contents in mice. (A)** β glucosidase activity. **(B)** Endoglucanase activity. **(C)** Exoglucanase activity. **(D)** Filter paper enzyme activity. **(E)** Xylanase activity. **(F)** Protease activity. **(G)** Amylase activity. **(H)** Lipase activity. “*” indicates a significant difference (*P* < 0.05), “***” indicates a significant difference (*P* < 0.001), “****” indicates a significant difference (*P* < 0.0001).


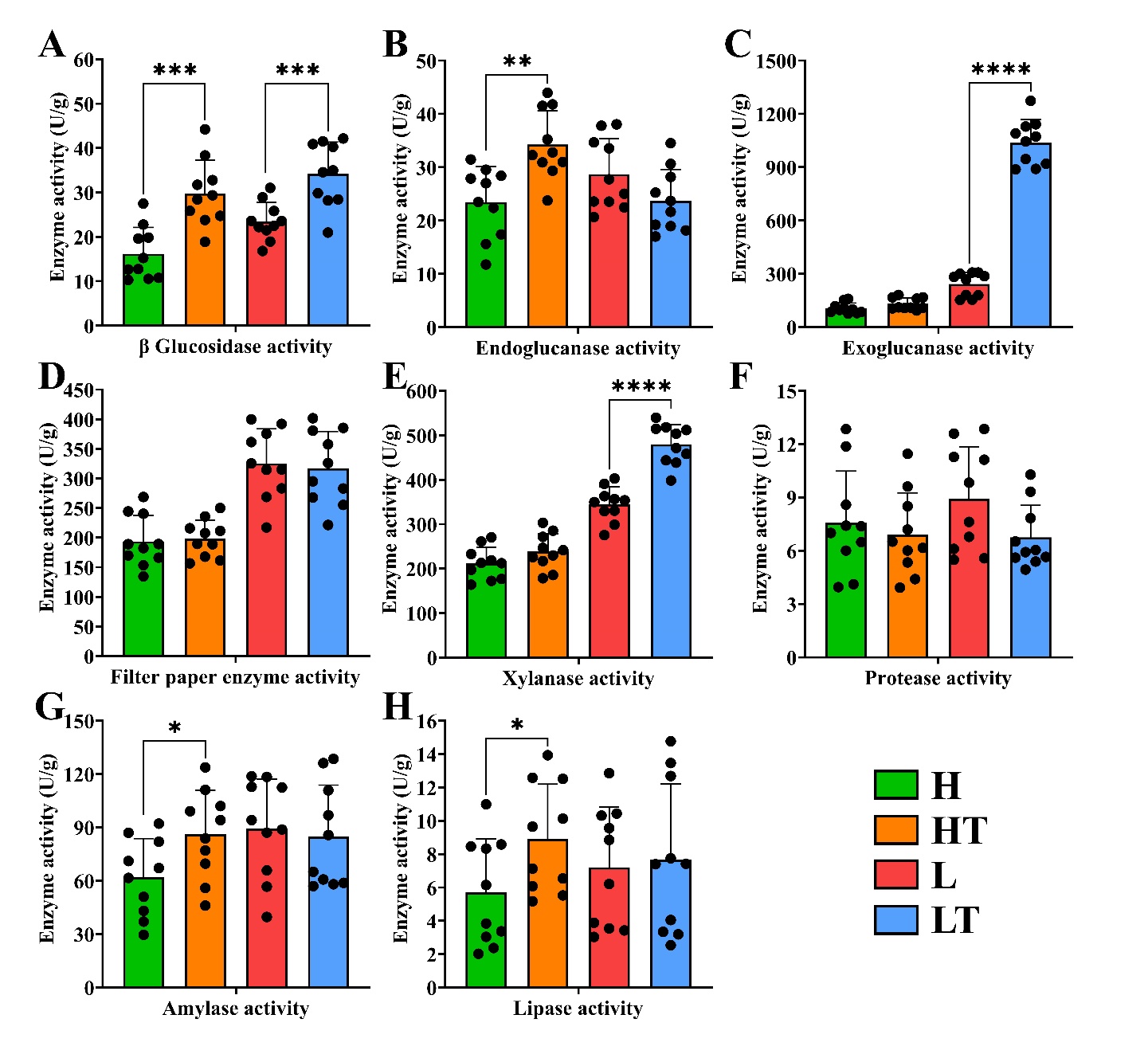


**Fig. S3 Digestive enzyme activity of ileum contents in mice. (A)** β glucosidase activity. **(B)** Endoglucanase activity. **(C)** Exoglucanase activity. **(D)** Filter paper enzyme activity. **(E)** Xylanase activity. **(F)** Protease activity. **(G)** Amylase activity. **(H)** Lipase activity. “*” indicates a significant difference (*P* < 0.05), “**” indicates a significant difference (*P* < 0.01), “***” indicates a significant difference (*P* < 0.001), “****” indicates a significant difference (*P* < 0.0001).


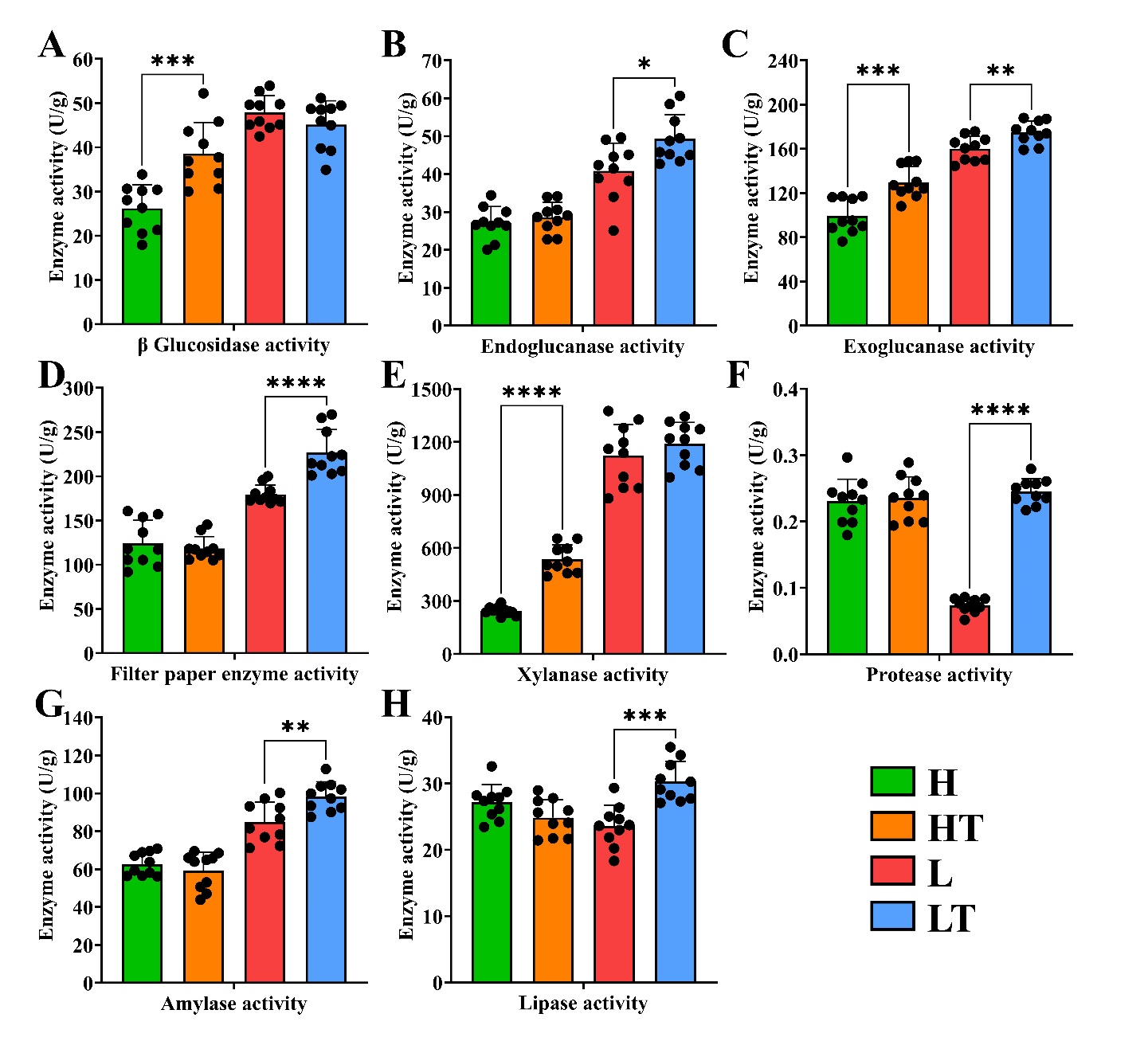


**Fig. S4 Digestive enzyme activity of colon contents in mice. (A)** β glucosidase activity. **(B)** Endoglucanase activity. **(C)** Exoglucanase activity. **(D)** Filter paper enzyme activity. **(E)** Xylanase activity. **(F)** Protease activity. **(G)** Amylase activity. **(H)** Lipase activity. “*” indicates a significant difference (*P* < 0.05), “**” indicates a significant difference (*P* < 0.01), “***” indicates a significant difference (*P* < 0.001), “****” indicates a significant difference (*P* < 0.0001).
